# Edge Contacts to Atomically Precise Graphene Nanoribbons

**DOI:** 10.1021/acsnano.3c00782

**Published:** 2023-08-14

**Authors:** Wenhao Huang, Oliver Braun, David I. Indolese, Gabriela Borin Barin, Guido Gandus, Michael Stiefel, Antonis Olziersky, Klaus Müllen, Mathieu Luisier, Daniele Passerone, Pascal Ruffieux, Christian Schönenberger, Kenji Watanabe, Takashi Taniguchi, Roman Fasel, Jian Zhang, Michel Calame, Mickael L. Perrin

**Affiliations:** † Transport at Nanoscale Interfaces Laboratory, Empa, Swiss Federal Laboratories for Materials Science and Technology, 8600 Dübendorf, Switzerland; ‡ Department of Physics, University of Basel, 4056 Basel, Switzerland; § nanotech@surfaces Laboratory, Empa, Swiss Federal Laboratories for Materials Science and Technology, 8600 Dübendorf, Switzerland; ∥ Department of Information Technology and Electrical Engineering, ETH Zurich, 8092 Zurich, Switzerland; ⊥ IBM Research − Zurich, 8803 Rüschlikon, Switzerland; # Max Planck Institute for Polymer Research, 55128 Mainz, Germany; 7 Quantum Center, ETH Zürich, 8093 Zürich, Switzerland; 8 Research Center for Electronic and Optical Materials, National Institute for Materials Science, 1-1 Namiki, Tsukuba 305-0044, Japan; 9 Research Center for Materials Nanoarchitectonics, National Institute for Materials Science, 1-1 Namiki, Tsukuba 305-0044, Japan; 10 Department of Chemistry, Biochemistry and Pharmaceutical Science, University of Bern, 3012 Bern, Switzerland; 11 Swiss Nanoscience Institute, University of Basel, 4056 Basel, Switzerland

**Keywords:** graphene nanoribbons (GNRs), electronic device, *h*-BN encapsulation, edge contacts, quantum dot, temperature-activated hopping

## Abstract

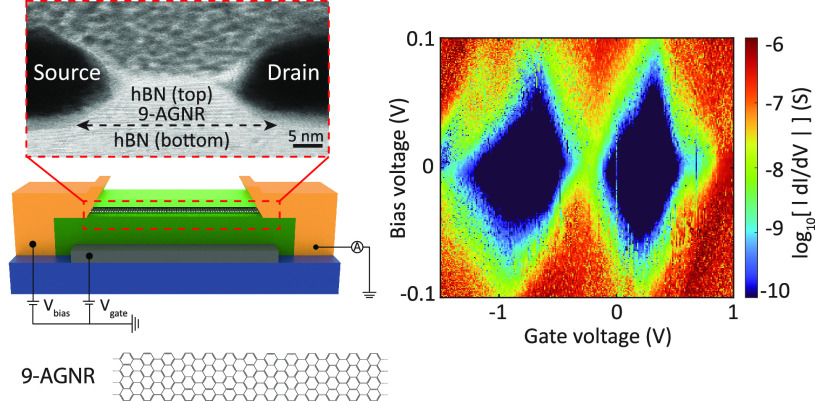

Bottom-up-synthesized graphene nanoribbons (GNRs) are an emerging class of designer quantum materials that possess superior properties, including atomically controlled uniformity and chemically tunable electronic properties. GNR-based devices are promising candidates for next-generation electronic, spintronic, and thermoelectric applications. However, due to their extremely small size, making electrical contact with GNRs remains a major challenge. Currently, the most commonly used methods are top metallic electrodes and bottom graphene electrodes, but for both, the contact resistance is expected to scale with overlap area. Here, we develop metallic edge contacts to contact nine-atom-wide armchair GNRs (9-AGNRs) after encapsulation in hexagonal boron-nitride (*h*-BN), resulting in ultrashort contact lengths. We find that charge transport in our devices occurs via two different mechanisms: at low temperatures (9 K), charges flow through single GNRs, resulting in quantum dot (QD) behavior with well-defined Coulomb diamonds (CDs), with addition energies in the range of 16 to 400 meV. For temperatures above 100 K, a combination of temperature-activated hopping and polaron-assisted tunneling takes over, with charges being able to flow through a network of 9-AGNRs across distances significantly exceeding the length of individual GNRs. At room temperature, our short-channel field-effect transistor devices exhibit on/off ratios as high as 3 × 10^5^ with on-state current up to 50 nA at 0.2 V. Moreover, we find that the contact performance of our edge-contact devices is comparable to that of top/bottom contact geometries but with a significantly reduced footprint. Overall, our work demonstrates that 9-AGNRs can be contacted at their ends in ultra-short-channel FET devices while being encapsulated in *h*-BN.

## I Introduction

Bottom-up-synthesized graphene nanoribbons (GNRs), with their chemically tunable electronic band structure, have received considerable attention in the past five years for their use in nanoelectronic devices, with a strong focus on field-effect transistors (FETs).^1−14^ For example, in 2017, Llinas *etal*.^15^ reported on the GNR-FETs with on/off ratios as high as 10^5^ at room temperature, while El Abbassi *etal*.^16^ reported on the quantum dot (QD) behavior in GNR-FETs at cryogenic temperatures. The overwhelming majority of FETs realized to date are based on armchair nanoribbons due to their excellent stability in ambient conditions, as well as their relative ease of synthesis and maturity.^9−11,15,16^ However, many other edge morphologies exist that have been shown to host intriguing physical phenomena.^17^ For example, GNR superlattices, in which topological boundary states are periodically coupled, lead to the formation of topologically protected states.^18−20^ Another appealing example is GNRs with zigzag edges, as they possess a net magnetic moment that leads to electronic edge states that couple ferromagnetically along the same edge and antiferromagnetically with the opposite edge.^21^ Exploring these GNRs for device application requires not only control over their chemical structure but also the preservation of their integrity upon device integration. In particular, due to their extremely small size, contacting GNRs remains a major challenge.^22^ Several approaches have been developed to date,^23^ relying on either metallic top contacts^4,14,15^ or bottom graphene electrodes^6,9,12,16,24^ and single wall carbon nanotubes^13^ electrodes. For metallic electrodes,^14,15^ photo- or electron-beam lithography is used, followed by a metallization step. However, this method can inadvertently cause polymer impurities to form at the contact junction and to inflict damage on the GNRs. Similarly, for bottom-fabricated graphene^12^ and carbon nanotube^13^ electrodes, polymeric residues on the electrode surface are challenging to avoid. In addition, for all of these contacting methods, the contact resistance is expected to scale with the area of overlap between the GNRs and the electrodes.

*h*-BN encapsulation combined with edge contacting has been widely used in the 2D material community to improve device performance by providing an atomically flat and electrostatically silent substrate,^25−31^ as well as a low contact resistance.^32^ Moreover, edge contacts have shown immunity to the contact-scaling problem, with performance that is independent of contact length.^30^ Finally, *h*-BN encapsulation is appealing, as it allows for air tightness,^33^ thereby preventing material degradation due to reaction with air, which is crucial for highly reactive materials that degrade due to the presence of oxygen. While for most 2D material devices the channel length is on the order of micrometers, GNR device’s channels are typically only tens of nanometers long at best, smaller than the length of GNRs themselves. To the best of our knowledge, no edge-contact approach has been reported for GNR-based devices.

Here, we report on the *h*-BN encapsulation and device integration of bottom-up-synthesized GNRs, contacted by metallic edge contacts. More specifically, we fabricate and electrically characterize short-channel FETs consisting of *h*-BN/9-AGNRs/*h*-BN heterostructures with channel lengths as short as ∼20–40 nm. The 9-AGNRs are contacted from their ends using metallic edge contacts, resulting in ultrashort contact lengths. A graphite flake is placed below the heterostructure to act as a gate.^32,34^ At room temperature, our devices exhibit on/off ratios as high as 3 × 10^5^, with on-state current up to 50 nA at 0.2 V. This demonstrates that the contact performance of our edge-contact devices is comparable to that of top contact geometries and better than bottom graphene and carbon nanotube contact. At cryogenic temperatures (9 K), our FETs exhibit QD behavior with the presence of CDs, with addition energies (*E*_add._) in the range of 16 to 400 meV, pointing toward the contacting of single or few GNRs per device. Interestingly, temperature-dependent measurements reveal that the charge transport mechanism is different between cryogenic and noncryogenic temperatures, with a crossover at 100 K. While at cryogenic temperatures electrons are transported resonantly through single levels of a quantum dot, at room temperature, temperature-activated hopping of charges occurs through the entire network of GNRs.

## II Results

### A Device Fabrication and Characterization

In this work, two types of devices are fabricated and characterized: (i) short-channel devices (SCFETs), in which the channel length (20–40 nm) is below or comparable to the average GNR length of 45 nm;^35^ here, single and/or few 9-AGNRs are expected to be in contact with both electrodes; (ii) long-channel devices (LCFETs), in which the channel length (1–2 μm) exceeds by far the GNR length. Here, no GNR is expected to bridge both electrodes, and transport can only occur through the GNR film via hopping between different GNRs.

**Figure 1:**
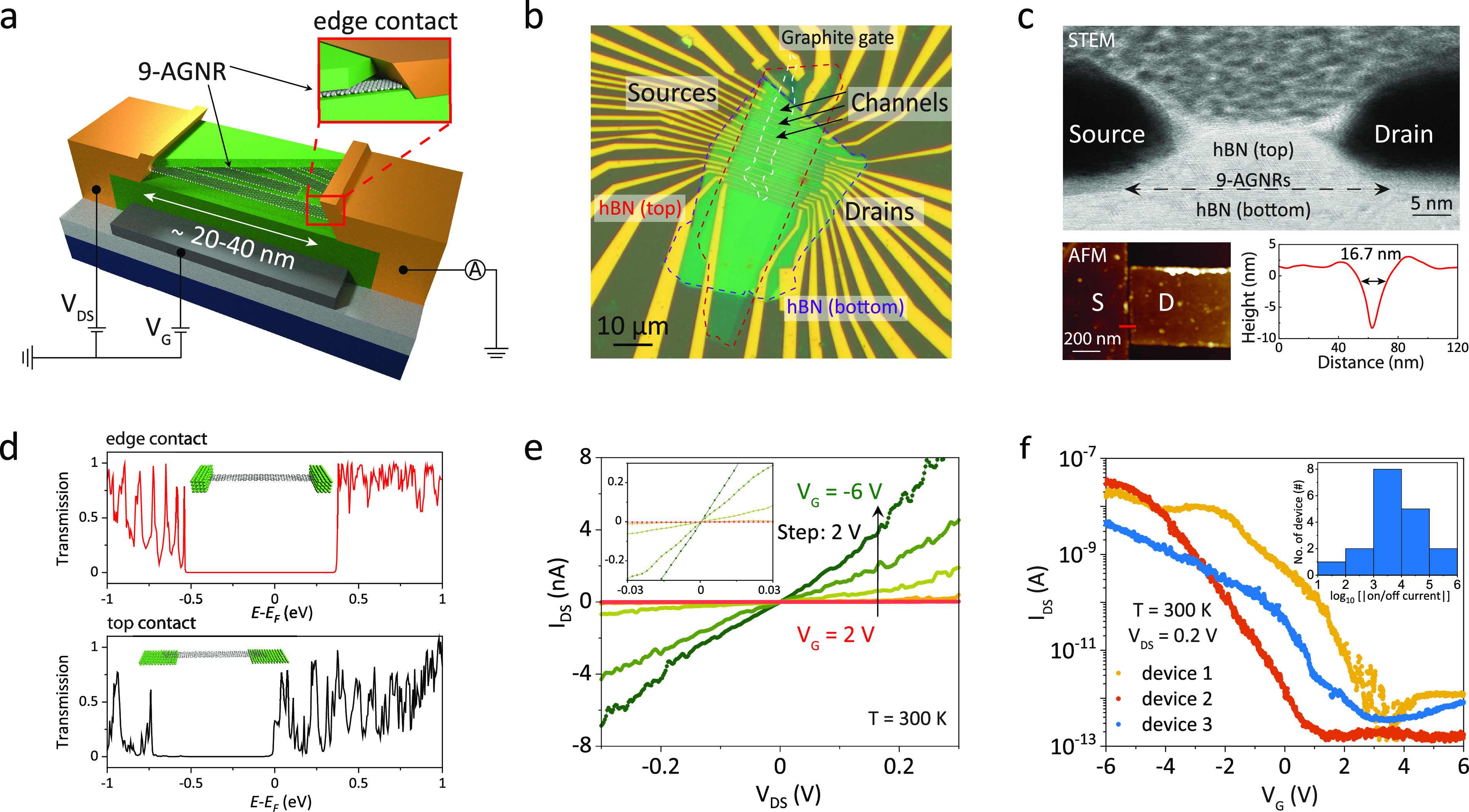
Device geometry and characterization. (a) Structure schematic of the 9-AGNRs SCFETs, including the measurement circuit. The film of 9-AGNRs is encapsulated between the top and bottom *h*-BN (light green), placed on a graphite gate (dark gray), and contacted by edge contacts (yellow). We measure the source–drain current (*I*_DS_) upon the application of source–drain bias (*V*_DS_) and gate voltage (*V*_G_). The top right inset shows a zoom-in configuration of the edge contact. (b) Optical micrograph of the measured device. The edges of the graphite gate (white), the bottom (purple) *h*-BN, and the top (red) *h*-BN are indicated by dashed colored lines. Source and drain contacts are labeled S and D, respectively. Their separation, labeled as channel, is below the resolution limit (∼20–40 nm). The channel width is ∼500 nm. (c) Top: STEM image showing a device cross-section. Arrows highlight the positions of the 9-AGNRs layer. Bottom left: AFM image of the channel region between the S and D contacts. Bottom right: line scan indicating the S and D contact separation (red line in the bottom left panel). (d) Computed transmission curves of edge contacts versus top contacts for chromium electrodes. The schematics illustrate the two contacting geometries: edge-contact (top) and top-contact geometry (bottom). (e) *I*_DS_–*V*_DS_ characteristics of representative SCFET devices at different *V*_G_ at 300 K. Inset: zoom-in of the low-bias range. (f) *I*_DS_ vs *V*_G_ curves at fixed *V*_DS_ = 0.2 V for 3 representative SCFET devices at 300 K. Inset: histogram of the on/off ratio for all measured SCFETs.

Figure 1a displays a schematic illustration of our SCFET based on *h*-BN-encapsulated 9-AGNRs. It consists of a film of 9-AGNRs sandwiched between two *h*-BN flakes (light green), with metallic edge contacts acting as the source (S) and drain (D) electrodes. The gate (G, dark gray) consists of a thin graphite flake located below the bottom *h*-BN (Supporting Information Figure S1). The top right inset of Figure 1a shows a close-up of the edge-contact configuration. In contrast to 2D materials, where the edge of the material is contacted, GNRs are quasi-1D objects due to their high geometrical anisotropy. Contacting a single GNR, therefore, occurs via an edge contact consisting of just a few atoms. The devices are fabricated as follows. First, the graphite gate and bottom *h*-BN flake are transferred to the device substrate following the transfer recipe of Wang *etal*.^32^ and Zomer *etal*.^27^ Nonaligned 9-AGNRs are then transferred using a polymer-free method from the growth substrate (Au/mica) to the device substrate.^36^ The top *h*-BN flake is placed on top of the 9-AGNRs after performing the thermal annealing of the device, as described in refs (5), (9), and (37). After placing the top *h*-BN, the integrity of the 9-AGNRs is verified using confocal Raman spectroscopy by inspection of the radial breathing-like mode (RBLM) peak (Supporting Information Figure S2).^38^ The edge contacts are then defined using a combination of electron-beam lithography (EBL) and reactive ion etching, followed by electron-beam-induced metal evaporation (3/20 nm Cr/Pd) (more details can be found in the Methods section and Supporting Information Figure S3). An optical micrograph of the measured device is shown in Figure 1b. The white, purple, and red dashed lines highlight the graphite gate, *h*-BN (bottom), and *h*-BN (top), respectively. The source, drain, and channels are labeled for clarity. The thicknesses of the top and bottom *h*-BN are determined by atomic force microscopy (AFM) to be ∼7 and ∼22 nm, respectively (Supporting Information Figure S1). The device geometry is assessed by using high-resolution scanning transmission electron microscopy (STEM), with a cross-section of a device shown in the top panel of Figure 1c. We note that the cross-section has been imaged after the electrical measurements. The edge contacts are visible, as well as the layered heterostructure. Within the resolution of the STEM image, there is no evidence of metallic particles or other impurities in the gap. From the image, we estimate the channel length to be about 35 nm. However, STEM imaging is a destructive approach that requires the preparation of a TEM lamella. As a complementary method, we use AFM to estimate the channel length. An AFM scan is presented in the bottom panels of Figure 1c, from which a metal separation of <∼20 nm on the surface of top *h*-BN is determined. The actual channel length *L*_C_ is estimated from the observed length deduced from the AFM measurement *L*_AFM_ and by taking into consideration the height of the top *h*-BN (7 nm) and the 45 deg angle generated by the RIE process, using *L*_C_ = *L*_AFM_ + 2*t*_*h*-BN_.^32^ (More details can be found in Supporting Information, Figure S4.) This AFM-based approach results in an estimate for channel length of *L*_C_ ≈ 31 nm, comparable to the value obtained from the STEM image.

Figure 1d shows computed transmission curves for edge contacts versus top contacts for chromium electrodes, obtained using density functional theory (DFT) combined with the nonequilibrium Green’s function formalism (NEGF). More details about the DFT+NEGF calculations are provided in the Methods section. The calculated bandgap is about 0.9 eV for the edge contacts, with the Fermi energy lying 0.54 eV away from the conduction band, well within the bandgap of the system. For the top contacts, the bandgap is reduced to 0.74 eV and the Fermi energy aligns with the first unoccupied orbitals that contribute to charge transport. The curves for the edge-contact geometry possess a comparable overall transmission compared to the top-contact, for both the occupied and unoccupied orbitals. The chemical contact between the ribbon and metallic surface, per se, would not ensure a favorable transmission function: it is necessary (and happens here) that the delocalized π-states of the ribbon maintain a good hybridization with the states of the metal contacts, providing suitable channels for electronic transport.

The devices are first characterized at a temperature of 300 K (more details can be found in the Methods). Current–voltage characteristics for different gate voltages are presented in Figure 1e for a representative device. As also supported by the zoomed-in plot in the inset, near-linear current–voltage characteristics are observed up to voltages of 0.3 V, indicative of a small Schottky barrier at the metal–GNR edge-contact interface. Additionally, we record the source–drain current (*I*_DS_) as a function of *V*_G_ at a fixed *V*_DS_ = 0.2 V. We find that all 18 devices show a p-type semiconductor behavior, consistent with previous reports.^2,9,15^ Throughout the devices, we find on-state currents up to 50 nA. In comparison, the latest devices with metal top contacts exhibit currents in the range of 5 to 300 nA under the same bias conditions.^4,14,15^ (More details can be found in Supporting Information Table S1.) Figure 1f presents three representative curves with the histogram of the on/off ratios of all measured SCFET devices. The maximum on/off ratio extracted from the *I*_DS_–*V*_G_ curves recorded on our SCFET devices is ∼3 × 10^5^, while the majority of the devices have an on/off ratio in the range of 10^3^–10^4^ (*V*_DS_ = 0.2 V, *V*_G_ = −6 to 6 V). As the current does not saturate in the applied gate range, it is likely that a larger on-current and hence a larger on/off ratio can be obtained by applying a wider range of *V*_G_ (see Supporting Information Figure S5 for *I*_DS_–*V*_G_ curves recorded on other SCFET devices). The subthreshold swing (SS), used to assess the FET’s switching efficiency, is calculated from the *I*_DS_–*V*_G_ curves. We estimate the effective SS values to be ∼468 mV/dec (more details are provided in Supporting Information Figure S6), comparable to GNR-FETs with a high-*k* dielectric gate oxide.^15^ Moreover, from the *I*_DS_–*V*_G_ curve, we extract the charge carrier field-effect mobility (μ_FE_), yielding values around ∼0.08 cm^2^ V^–1^ s^–1^ (more details are provided in Supporting Information Figure S7).

### B Electron Transport Characteristics in 9-AGNRs SCFETs

**Figure 2:**
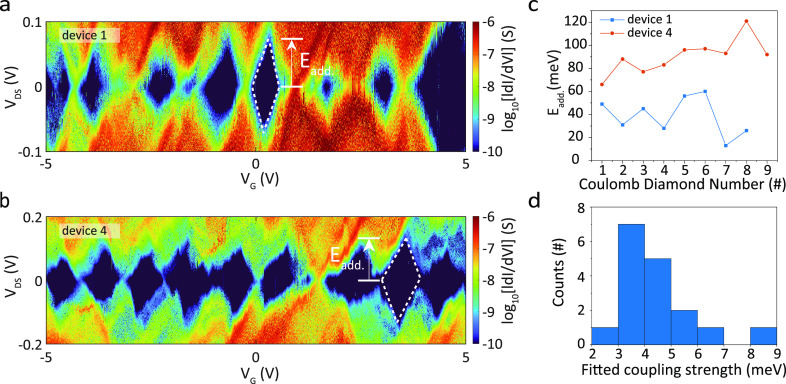
Quantum dot behavior at low temperature (9 K). Differential conductance d*I*/d*V* maps as a function of *V*_DS_ and *V*_G_ on a logarithmic scale recorded on (a) device 1 and (b) device 4. Dashed white lines guide the Coulomb diamond edges, and the height of the Coulomb diamonds are labeled as *E*_add._. (c) Overview graphs of the corresponding *E*_add._ as a function of the Coulomb diamond number extracted from devices 1 and 4. (d) Histogram of the fitted coupling strength Γ of all 17 crossing points obtained from the Breit–Wigner (BW) model fittings.

Next, to investigate the charge transport properties of the devices at low temperature, we perform electrical measurements on 18 SCFET devices (labeled 1, 2, 3, etc.) at a cryogenic temperature of 9 K, where 15 of these devices were functional. Figure 2a and 2b present the differential conductance (d*I*/d*V*) as a function of *V*_DS_ and *V*_G_ on a logarithmic scale recorded on devices 1 and 4, respectively. The two plots exhibit multiple diamond-shaped areas in which charge transport is blocked. This indicates the formation of quantum dots in the SCFETs. As a consequence, the energies of the transport channels are quantized to discrete values. The regions in the differential conductance plot in which electron transport is blocked are referred to as CDs. Here, the charge carriers do not possess sufficient energy to be transported through the device. The edges of these CDs correspond to the onset of resonant charge transport from the source to the drain electrode, occurring when an energy level of the GNR QD enters the bias windows and single-electron tunneling (SET) takes place. Both the blocking and the SET regime are characteristic of charge transport through quantum dots and indicate that the 9-AGNRs in our SCFETs behave as such (more data can be found in Supporting Information Figure S8).

Throughout the measured samples, we observe different types of behavior: (i) the edges of the Coulomb blockade regions meet and cross at zero bias voltage, where the electrochemical potential of the QD is aligned with the electrochemical potential of the two electrodes. This corresponds to the situation in which a single or a few similar 9-AGNRs have been contacted by both electrodes and contribute to electron transport. In this scenario, as exemplified in both devices 1 and 4, the electronic structure of the single QD can be assessed, as will be discussed later. (ii) the Coulomb blockade regions are diamond-shaped, but no crossing points are observed between the CDs at zero bias, as observed for device 4 at a gate voltage of −1 V and shown in Figure 2b. This can have different causes: (a) multiple 9-AGNRs with different energy levels are contacted in series; (b) a charged impurity is present close to the QD.^39,40^ In device 4, the latter scenario is more plausible as the neighboring CDs do have well-defined crossing points. (iii) Several nonclosing CDs are overlapping. Here, multiple GNRs are connected in parallel and possibly also in series (see Supporting Information Figure S8).

To study the electronic structure of the quantum dots, we extracted the energy spacing between the different transport levels. For this, the size of the CDs for which crossing points are present (type i) are analyzed and the values of the addition energies (*E*_add._) are extracted (as an example, white dashed lines and arrows are shown in the differential conductance maps in Figure 2a and 2b). Figure 2c displays the *E*_add._ as a function of the Coulomb diamond number for two representative SCFET devices (more data can be found in Supporting Information Figure S9). Overall, the addition energies vary significantly within a single device but also from device to device, in a total range between 16 and 400 meV. The intradevice variability is a direct reflection of the electronic structure of the QD, with, depending on the charge state, contributions to the addition energy from the charging energy and/or the quantum mechanical level splitting. The interdevice variation is attributed to multiple effects: (1) The contacted GNRs may not all have the same length,^35^ leading to different confinement potentials. (2) The local electrostatic potentials and screening effects can be dependent on the number of electrons hosted by the dot. (3) The GNRs in different devices may be in different charge states.

To estimate the quality of the contact between the GNR and metallic electrodes, we extracted the coupling of the QD to the leads. From the differential conductance map (type i), we extract a line cut at zero bias and fit the resonances with the Breit–Wigner (BW) model for resonant transport through a single-lifetime-broadened transport level.^41^ This provides us with the tunnel coupling strength (Γ) between a single GNR and the leads (see Methods). Here, for simplicity, we assume that the two coupling strengths are symmetric. In total, we collect the coupling strength from 17 crossing points originating from six different devices, of which a histogram is shown in Figure 2d. From the fitting results, we find an average coupling of 4.43 ± 4.22 meV and a maximum of 8.65 meV. To exclude that the resonances are purely broadened by temperature, we fit our data to thermally broadened resonances (see Methods).^41^ We find that the average fitted temperature is ∼30 K, which is significantly higher than the cryostat temperature (9 K). This indicates that the broadening of the resonances observed in our measurement is the result of the combined effect of hybridization with the electrode and temperature (see more details in Supporting Information Figure S10). In addition, we also performed temperature-dependent measurements on our SCFET devices. In Figures S11 and S12 of the Supporting Information, we show that the resonances gradually smear out with increasing temperature and are completed washed out at temperatures above 120 K.

### C Electron Transport Characteristics in 9-AGNRs LCFETs

**Figure 3:**
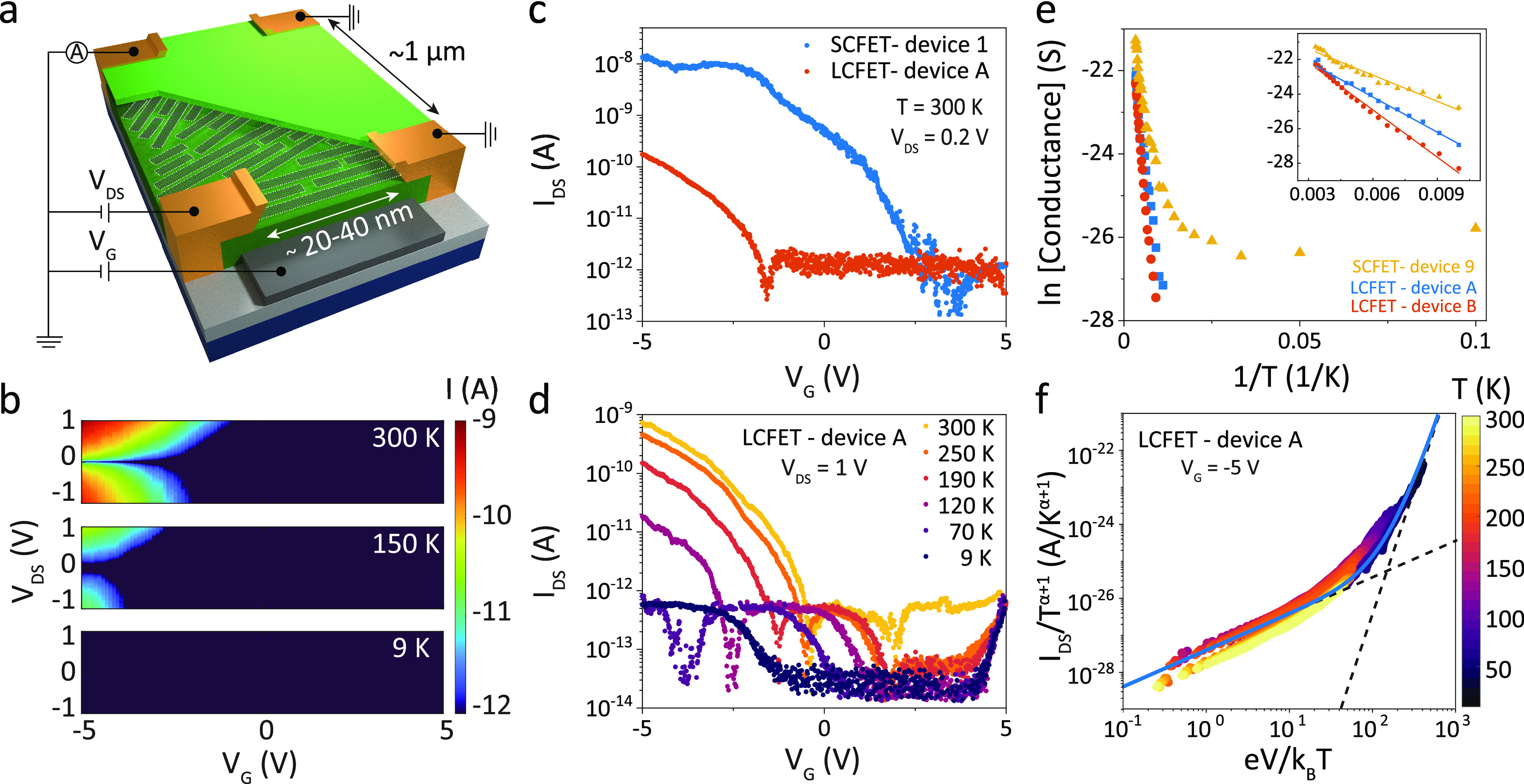
Charge transport in GNR networks. (a) Schematic illustration of long-channel FETs (LCFETs) with the measurement circuit. The film of 9-AGNRs is encapsulated between *h*-BN (light green), placed on a graphite gate (dark gray), and contacted from the side by edge contacts (yellow) separated by a larger distance than the average 9-AGNR length, in the range of μm. We measured the *I*_DS_ upon application of *V*_DS_ and *V*_G_. (b) Maps of *I*_DS_ as a function of *V*_DS_ and *V*_G_ recorded on device A at various temperatures (only 9, 150, and 300 K, shown here; see more details in Supporting Information Figure S16). (c) Transport characteristics of SCFET device 1 and LCFET device A as a function of *V*_G_, recorded at 300 K (*V*_DS_ = 0.2 V). (d) Transport characteristics of device A at fixed *V*_DS_ = 1 V as a function of *V*_G_, recorded at various temperatures. (e) Arrhenius plot of the conductance recorded from three different devices at *V*_DS_ = 0.4 V and *V*_G_ = −5 V as a function of 1/*T*. The inset is linear fittings within 300 to 100 K for these three different devices. (f) The temperature-dependent current–voltage characteristics at *V*_G_ = −5 V as scaled current I/*T*^α+1^ versus scaled bias voltage eV/*k*_B_*T*.

In the previous section, we focused on SCFET devices in which one or a few GNRs are in contact with both the source and drain electrodes simultaneously. However, GNRs are grown in dense films (see Supporting Information Figure S13), and such films have been shown to form a network that conducts charges over length scales exceeding the dimension of a single GNR. In these networks, the current is mainly driven by temperature-activated hopping between localized sites and polaron-assisted tunneling.^10^ Here, we hypothesize that, in addition to the charge transport through the quantum dots, a parallel conductance channel through the GNR network opens up at high temperatures. To investigate this behavior, we studied charge transport between adjacent devices. Here, the electrode separation is micron-sized, more than an order of magnitude larger than the average GNR length.^35^ A schematic of these devices, in the following referred to as the LCFET, is presented in Figure 3a. Similar to the SCFET devices, the graphite flake serves as a gate. The top panel of Figure 3b presents a color-coded map of the current *I*_DS_ as a function of *V*_DS_ and *V*_G_ recorded at 300 K on device A (the 9-AGNR LCFET devices are labeled A, B, C, etc.). Similar to the SCFET characteristics shown in Figure 1f, clear p-type semiconductor behavior was observed. Figure 3c presents a typical gate trace obtained for both SCFET and LCFET device geometries for *V*_DS_ = 0.2 V. The plot shows that *I*_on_ at *V*_G_ = −5 V for the LCFETs are typically 2 to 3 orders of magnitude lower than the SCFETs. Nevertheless, *I*_on_ can be significantly increased to ∼nA for *V*_DS_ = 1 V and *V*_G_ = −5 V (see Supporting Information Figure S14 for linecuts, current versus gate traces, obtained from the current map of device A for various bias voltages; more data from other LCFET devices can be found in Supporting Information Figure S15).

To investigate in more detail the transport mechanism through the GNR network, maps of *I*_DS_ as a function of *V*_DS_ and *V*_G_ for various temperatures are recorded. For two devices (A and B), we studied the evolution of the charge transport properties with decreasing temperature from 300 K to 9 K in steps of 10 K, with some examples shown in Figure 3b. A more extensive version of the data set is presented in Supporting Information Figures S16 and S17. Figure 3b shows that when lowering the temperature, while the p-type behavior is conserved, the current decreases and eventually drops below the measurement limit of our system at 9 K. This behavior is emphasized when plotting the current versus gate voltage for device A at fixed *V*_DS_ = 1 V for various temperatures (Figure 3d). The on-state current drastically drops with a decrease in temperature until it reaches the measurement limit at 9 K.

We plot the data for fixed *V*_DS_ = 0.4 V and *V*_G_ = −5 V in an Arrhenius plot, as shown in Figure 3e. The Arrhenius equation (ln(|*G*|) , where *T* is the bath temperature) is used to analyze temperature-activated transport.^42^ For both devices A and B, the logarithm of the current decreases linearly in the Arrhenius plot until reaching the measurement limit of the system. The inset zooms in the low 1/*T* range (high-temperature regime). For both devices, the data are fitted to the Arrhenius equation in the temperature range 100–300 K. As a comparison, we plot in the same fashion the current through the SCFET device 9. For this device, a similar decrease in conductance is observed in the high-temperature regime until the plot starts to deviate around *T* = 100 K and eventually flattens out around *T* = 50 K. In the high-temperature regime, the excellent fit suggests that temperature-activated hopping of charge carriers between localized sites is indeed the dominant transport mechanism, for both the SCFET and the LCFET devices, with activation energies (*E*_act_) of 58.28 meV (device A), 78.44 meV (device B), and 43.39 meV (device 9). As the activation energy in the SCFETs and LCFETs under the same biasing conditions are of similar magnitude, we conclude that the same transport mechanism is at play between 100 and 300 K, namely, temperature-activated hopping. On the other hand, in the lower temperature range, the SCFETs and LCFETs behave very differently. While for the LCFETs the current decreases to the measurement limit, the SCFET device exhibits a transition around 100 K and flattens out around 50 K. Then, the conductance becomes temperature independent. This temperature range corresponds to the formation of quantum dots, as is evident from the differential conductance maps, for which the temperature dependence is expected to be limited. We, therefore, conclude that throughout the downward temperature sweep of the SCFET, the charge transport mechanism changes from hopping through the film of GNRs to transport through the QD (more details can be found in Supporting Information Figure S18).

Recently, it was shown that, for the network of GNR, hopping (thermally assisted tunneling of charge carriers between localized sites), as described by the semiclassical Marcus electron transfer theory, provides only a limited description of charge transport.^10,43^ While hopping transport is present when either temperature or bias voltage provides enough energy to overcome the classical barrier, it is expected to vanish for temperatures approaching absolute zero. However, in disordered semiconductors with a high charge carrier density, even at low temperatures, finite conductance has been shown to exist. This has been attributed to polaron-assisted tunneling through the classical barrier.^10,43^ Both mechanisms therefore coexist, and the current obeys a scaled master curve for current–voltage characteristics with bosonic excitations in 1D.^44^ Here, for low-bias voltages, charge transport obeys a power-law dependence on temperature. In the high-bias limit, transport becomes temperature independent. The two limits are illustrated in Figure 3f by the dashed lines. A crossover between the two occurs when the potential electrical energy of the carriers for a single hop^45,46^ is equal to the thermal energy of the carrier.

To identify whether hopping and polaron-assisted tunneling are also at play in our devices, we plot in Figure 3f the temperature-dependent current–voltage characteristics at negative gate voltage (*V*_G_ = −5 V) as scaled current I/*T*^α+1^ versus scaled bias voltage eV/*k*_B_*T*. From the excellent fit (see Methods section and Supporting Information Figure S19), we conclude that also in our case the charge transport mechanism for high charge-carrier density is a combination of semiclassical electron hopping and polaron-assisted tunneling through the classical barrier. However, for positive gate voltages where the currents are low, charge transport only occurs in the high-bias limit. This suggests that for such low charge carrier densities the activation energy is larger and temperature is not able to drive transport (see Figure S20 of the Supporting Information).

## III Discussion

Similarly to previous work on charge transport through GNR networks, we have extracted the field-effect mobilities for our SCFET devices, with values up to ∼0.08 cm^2^ V^–1^ s^–1^. This is more than 1 and 3 orders of magnitude higher than reported in ref (10) (∼10^–3^ cm^2^ V^–1^ s^–1^) and ref (47) (∼10^–5^ cm^2^ V^–1^ s^–1^), respectively. The extracted μ_FE_ is among the highest values reported in FETs, with hopping as the dominant charge transport mechanism^48^ (more details are provided in Supporting Information Figure S7). We note that for both the SCFET and LCFET devices the charge transport mechanism at 300 K is temperature-activated hopping transport, making the comparison of the mobility with other work on GNR networks possible. This higher mobility may be due to the *h*-BN substrate, which offers an atomically flat and trap-free interface, along with a small lattice mismatch and protection of the GNRs from the environment. However, care should be taken, as fringe currents are present in organic thin films since the electrically contacted film extends beyond the geometrically defined transport channel between the source and drain, which may lead to the overestimation of the electron mobility.^49^

In this study, we utilized 9-AGNRs that are stable under ambient conditions to demonstrate the feasibility of *h*-BN encapsulation as well as edge-contact fabrication. Edge contacts provide a scalable strategy to fabricate transistors with a contact length down to the atomic scale, offering promising prospects for ultimately scaled, high-density electronic devices. As *h*-BN encapsulation has been shown to be airtight,^33^ our strategy provides a possible pathway to protect the GNRs against degradation due to reaction with the environment. However, no appropriate methods are available to date for transferring reactive GNRs to devices. This would require the development of dry-transfer methods avoiding the use of any chemicals, ideally performed under controlled atmospheric conditions or even vacuum conditions.^50^ Alternatively, chemical methods for protecting the reactive sites could be employed.^51^

## IV Conclusion

In conclusion, we successfully fabricated edge contacts for *h*-BN-encapsulated 9-AGNRs, with source–drain separations as small as ∼20 nm. Our SCFET devices show QD behavior at 9 K, characteristic of only a single or few 9-AGNRs contributing to transport. We demonstrate that, for increasing temperature, charge transport through the GNR network starts to dominate above 100 K, with as the main mechanism temperature-activated hopping and polaron-assisted tunneling. In addition, at room temperature, the 9-AGNR SCFETs show a p-type semiconductor behavior with a maximum on/off ratio of up to 3 × 10^5^. Our approach offers a promising way to create good electrical contacts while at the same time reducing the device footprint by bringing the contact length down to the atomic scale. Moreover, *h*-BN encapsulation is an appealing strategy for providing air tightness and preventing material degradation. This approach may enable the integration into devices of GNRs that are unstable under ambient conditions and exhibit topologically^18^ and magnetically^52^ nontrivial quantum phases.

## V Methods

### On-Surface Synthesis of 9-AGNRs and Device Integration

9-AGNRs are synthesized from 3′,6′-diiodo-1,1′:2′,1′-terphenyl (DITP).^35^ Using Au(111)-/mica leads to nonaligned GNRs.^53,54^ Au(111)/mica growth substrates (Phasis, Switzerland) are cleaned in ultrahigh vacuum by two sputtering/annealing cycles: 1 kV Ar^+^ for 10 min followed by annealing at 470 °C for 10 min. Next, the precursor monomer DITP is sublimed onto the Au surface from a quartz crucible heated to 70 °C, with the growth substrate held at room temperature. After deposition of 1 monolayer DITP, the growth substrate is heated (0.5 K/s) to 200 °C with a 10 min holding time to activate the polymerization reaction, followed by annealing at 400 °C (0.5 K/s with a 10 min holding time) to form the GNRs via cyclodehydrogenation. The average 9-AGNR length is around 40–45 nm.^35^

### Device Fabrication

Graphite/*h*-BN/9-AGNRs/*h*-BN heterostructure preparation begins with mechanical exfoliation of graphite (NGS Trading & Consulting GmbH) and *h*-BN flakes (National Institute for Materials Science, Japan) on substrates from bulk materials, and then the thin graphite and the bottom *h*-BN are first stacked using a micromanipulator (hq Graphene 2D heterostructure transfer system) to form the graphite gate. After that, using our chemical vapor deposition system, an annealing step is performed at 300 °C with H_2_/Ar: 35/200 sccm for 3 h to improve the quality of the heterostructures by reducing interfacial bubbles, contaminants, etc. We note that no material is deposited in this step. Then, the transfer of the 9-AGNRs is done by using a polymer-free method followed by a thermal annealing step as described in refs (5, 9, 36, and 37). Finally, we use the same precise transfer method to stack the top *h*-BN. An oxygen plasma step is performed to remove the GNRs outside the area covered by the top *h*-BN. Next, the edge contacts are defined by EBL with 90 nm poly(methyl methacrylate) (PMMA) and reactive ion etching (RIE) (CHF_3_/O_2_: 40/4 sccm, 60 mTorr, 60 W, etching rate: 28 nm/min). We deposit 3/20 nm Cr/Pd using an e-beam evaporator for edge contacts, and the pattern is lifted off using acetone for 45 min. After that, the second EBL and metal deposition (5/65 nm Cr/Au) are performed for contact pads.

### DFT Calculations

Our strategy to compute the electronic transport properties is composed of several steps. First, a Cr(100) slab is prepared with periodic boundary conditions in all directions with the surface layers allowed to relax and the central layers fixed at the bulk positions. A 9-AGNR bridges the two surfaces so that, thanks to periodic boundary conditions, a cyclic geometry is obtained. The distance between the two surfaces is allowed to adapt to the ribbon length. The termini of the ribbon (with a zigzag profile) are not saturated with hydrogens to allow chemisorption to the surface. It is known that CH termination leads to localized end states in the bandgap. We use the Gaussian basis set-based code CP2K.^55^ The electronic wave function is expanded in localized basis functions, and the electronic structure is described using density functional theory with the GGA PBE exchange and correlation potential, with D3 phenomenological corrections for the van der Waals interactions.^56^ The atoms are displaced using the BFGS optimization algorithm until all components of the forces are smaller than 5 × 10^–4^ au. Once the geometry has been optimized with CP2K, we applied the NEGF procedure described previously^57^ to compute the electronic transport. This procedure is based on modeling a scattering region formed by the ribbon and the contacted part of the leads explicitly, where the Hamiltonian and overlap matrix are extracted from the DFT calculation. The corresponding self-energy and Green’s functions are computed in the lead region using a periodically repeated metal geometry. This self-energy and the corresponding Green’s function are then used to compute the transmission function.

### Device Characterization

Exfoliated flakes are optically screened using an optical microscope (Zeiss Axio imager M2m). The thickness of *h*-BN layers and the source–drain separation are characterized by using AFM in the tapping mode (Bruker Icon3 AFM). The STEM image is gained from a FEI Titan Themis 3510. Raman spectroscopy (WITec Alpha300 R) is used to confirm the successful GNR transfer.^58^ The presence of the RBLM after transfer to the device substrate is a strong indicator of the GNRs’ integrity upon device integration.^38^

### Electronic Measurements

All electronic measurements are performed under vacuum conditions (<10^–6^ mbar). The devices are measured in a commercially available probe station (Lake Shore Cryogenics, model CRX-6.5K) at various temperatures (9–300 K). A data acquisition board (ADwin-Gold II, Jäger Computergesteuerte Messtechnik GmbH) is employed to apply the bias and gate voltages and read the voltage output of the *I*–*V* converter (DDPCA-300, FEMTO Messtechnik GmbH).

### Electronic Coupling Fitting

In the Breit–Wigner model, the peak shape is described by^41^
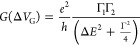
1with Γ = Γ_1_ + Γ_2_ and the QD level detuning:
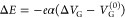
2where *V*_G_^(0)^ is the position of the resonance. Here, α is the gate coupling of the gate to the QD, described by using the following relation:
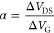
3

### Thermal Broadening Fitting

The conductance of a thermally broadened level is described as follows:^41^
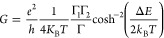
4with *k*_B_ being the Boltzmann constant and *T*, the bath temperature.

### GNR Network Transport Fitting

The hopping rate equation and the bias current are described as follows:^10,43^
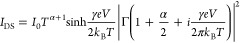
5where γ^–1^ is the hop number and α is a scaled version of the Kondo parameter. Two specific regimes can be identified:

(i) In the high-voltage regime, with β = α + 1, hopping transport is present and the current is given by
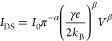
6
(ii) In the low-voltage regime, polaron-assisted tunneling takes over:
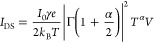
7

## Supporting Information Available

Figures of *h*-BN flake preparation; Raman characterization of encapsulated GNRs; device fabrication; schematic cross-section of the channel after the RIE process; gate sweeps recorded on SCFETs; subthreshold swing extraction for SCFETs; charge carrier field-effect mobility extraction for SCFETs; quantum dot behavior in SCFETs at 9 K; additional energy extraction; fitting of electronics coupling; temperature-dependent transport characteristics; temperature-dependent differential conductance maps; scanning tunneling microscope image of 9-AGNRs grown on Au/mica under ultra-high-vacuum conditions; bias-dependent transport characteristics of LCFETs; gate sweeps recorded on LCFETs; temperature-dependent current maps of device A; temperature-dependent current maps of device B; temperature-dependent current maps of device 9; linear fit for determining the scaled Kondo parameter α; gate dependence of hopping and polaron-assisted tunneling; table showing comparison of on-state current between this work and previous GNR devices (PDF)
