# Supplementary material for: Edge Contacts to Atomically Precise Graphene Nanoribbons
Source: ACS Nano. 2023 Aug 14;17(19):18706–15. doi: 10.1021/acsnano.3c00782 (PMC10569104; doi:10.1021/acsnano.3c00782)
Supplement: Supplementary file 1 — nn3c00782_si_001.pdf [file nn3c00782_si_001.pdf]

Supplementary Information for Edge Contacts to Atomically Precise Graphene Nanoribbons

Wenhao Huang,[1,2] Oliver Braun,[1,2] David I. Indolese,[2] Gabriela Borin Barin,[3] Guido Gandus,[3,4] Michael Stiefel,[1] Antonis Olziersky,[5] Klaus Müllen,[6] Mathieu Luisier,[4] Daniele Passerone,[3] Pascal Ruffieux,[3] Christian Schönenberger,[2] Kenji Watanabe,[7] Takashi Taniguchi,[8] Roman Fasel,[3,9] Jian Zhang,[1,*] Michel Calame,[1,2,10,†] and Mickael L. Perrin[4,1,11,‡]

[1] *Transport at Nanoscale Interfaces Laboratory, Empa, Swiss Federal Laboratories for Materials Science and Technology, 8600 Dübendorf, Switzerland*

[2] *Department of Physics, University of Basel, 4056 Basel, Switzerland*

[3] *nanotech@surfaces Laboratory, Empa, Swiss Federal Laboratories for Materials Science and Technology, 8600 Dübendorf, Switzerland*

[4] *Department of Information Technology and Electrical Engineering, ETH Zurich, 8092 Zurich, Switzerland*

[5] *IBM Research – Zurich, 8803 Rüschlikon, Switzerland*

[6] *Max Planck Institute for Polymer Research, 55128 Mainz, Germany*

[7] *Research Center for Functional Materials, National Institute for Materials Science, 1-1 Namiki, Tsukuba 305-0044, Japan*

[8] *International Center for Materials Nanoarchitectonics, National Institute for Materials Science, 1-1 Namiki, Tsukuba 305-0044, Japan*

[9] *Department of Chemistry and Biochemistry, University of Bern, 3012 Bern, Switzerland*

[10] *Swiss Nanoscience Institute, University of Basel, 4056 Basel, Switzerland*

[11] *Quantum Center, ETH Zürich, 8093 Zürich, Switzerland*

I. DEVICE FABRICATION AND RAMAN CHARACTERIZATION

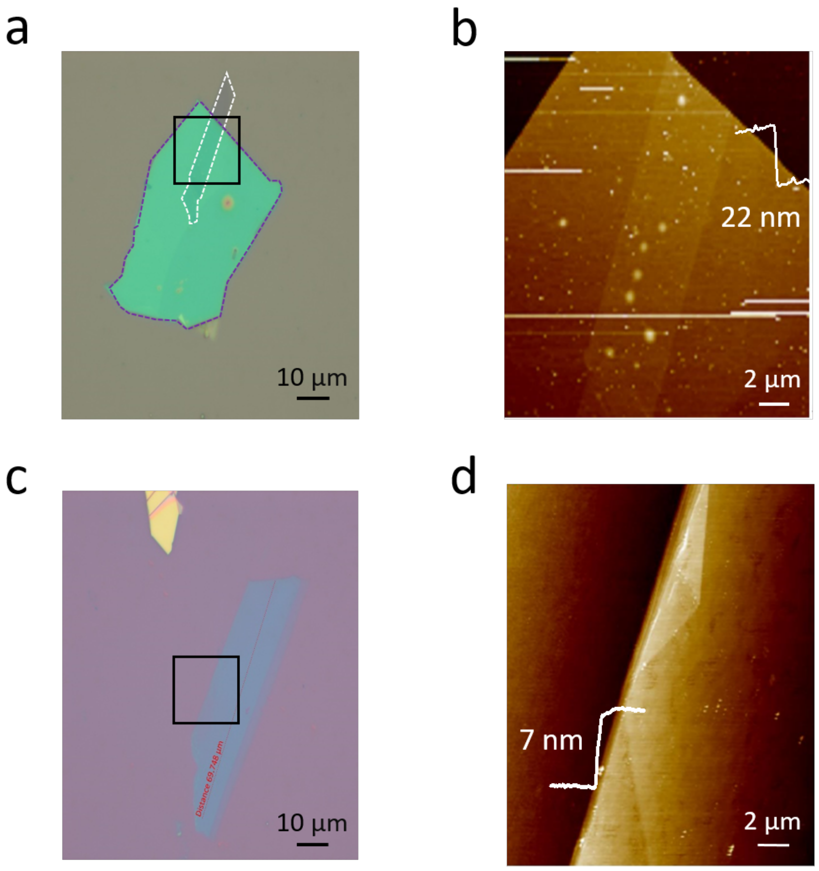

Figure S1. *h*−**BN flake preparation. a)** Optical image showing a thin graphite flake and the bottom hexagonal Boron Nitride (*h*-BN) flake. The edge of the graphite and *h*-BN are highlighted by a dashed white and purple lines, respectively. A black box indicates the atomic force microscopy (AFM) scan area shown in b). **b)** AFM map, including a height profile of the bottom *h*-BN obtained at the location indicated by the thick white line. The thickness is determined to be ∼22 nm. **c)** Optical image of the top *h*-BN flake. A black box indicates the atomic force microscopy (AFM) scan area shown in d). **d)** AFM map, including a height profile of the top *h*-BN obtained at the location indicated by the thick white line. The thickness is determined to be ∼7 nm.

To verify the quality of the 9-AGNRs, we obtained Raman spectra using a confocal Raman microscope (WITec, Alpha 300 R) for both the before-transfer (on Au substrate)

* jian.zhang@empa.ch
† michel.calame@empa.ch
‡ mickael.perrin@ee.ethz.ch

and after-transfer samples (on h-BN substrate) within a vacuum chamber. An excitation laser with a wavelength of 785 nm is used for the Raman measurement. Both spectra exhibit several distinct peaks: the radial breathing-like mode (RBLM) at 313 cm^{-1}, which is consistent with previous work,[1, 2] an edge-related mode at 1230 cm^{-1}, with a D-mode at 1335 cm^{-1} and a G-mode at 1595 cm^{-1}. The presence of these modes is indicative of high-quality 9AGNRs.It is worth noting that we observe a large background signal on the spectrum obtained from the GNRs/h-BN sample, which can be attributed to the presence of an additional h-BN signal.[3] Nevertheless, it is important to mention that both spectra exhibit typical Raman characteristic peaks of GNRs, indicating that GNRs still keep their integrity after the transfer.

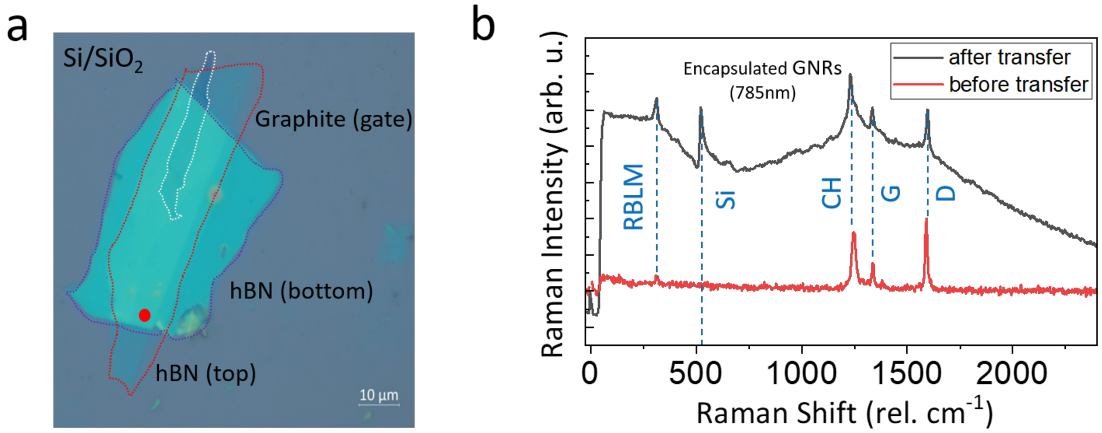

Figure S2. **Raman characterization of encapsulated GNRs.** **a)** Optical image of the graphite/h-BN/9-AGNR/h-BN heterostructure. The dashed white, purple, and red lines highlight the graphite, bottom h-BN, and top h-BN, respectively. **b)** The Raman spectrum before and after transfer. (The Raman after transfer was recorded at the red spot in Fig. S2a.

For the heterostructure preparation, we follow the transfer recipe which is described in previous works.[4, 5] The pick-up process is performed using PDMS-backed polycarbonate (PC) stamps. PC shows a good adherence to h-BN for 30 °C $\leq$ T $\leq$ 60 °C. After the successful pick-up of the top h-BN layer, the thin graphite flake is picked up using the van der Waals interaction between the graphite and h-BN. Then, the heterostructure is placed on a pre-patterned Si^{++}/SiO_2 (285 nm) wafer at T = 150 °C. The PC is dissolved in dichloromethane (DCM). A CVD annealing step is performed at 350 °C with H_2/Ar:

35/200 sccm for 3 hours to improve the quality of the heterostructures by reducing interfacial bubbles, contaminants etc. Next, 9-AGNRs are transferred from their growth substrate to the silicon-based target substrate with the graphite gate and the bottom *h*-BN by using a polymer-free method followed by a thermal annealing step as described in Ref.[6–9]. Finally, the same stacking process is used for the top *h*-BN transfer. An oxygen plasma step is performed to remove the GNRs which are out of the top *h*-BN. Next, the edge-contact electrodes are defined by electron beam lithography (EBL) with 950K PMMA and reactive ion etching (RIE) (CHF_3/O_2: 40/4 sccm, 60 mTorr, 60 W, the etching rate: 28 nm/min). The edge contacts are then deposited using an e-beam evaporator (3/20 nm Cr/Pd) and the pattern is lifted off using acetone for 45 min. After that, a second EBL step and metal deposition (5/65 nm Cr/Au) are performed for the contact pads.

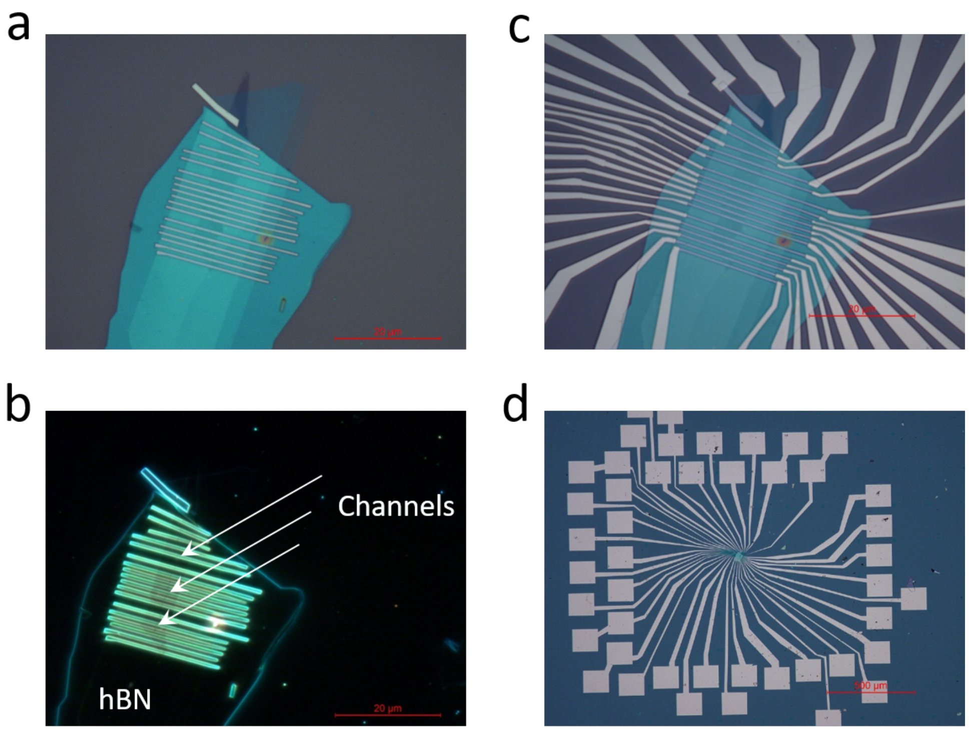

Figure S3. **Device fabrication. a)** Optical image of the fabricated device after the first electron beam lithography (EBL), the reactive ion etching (RIE) and the metal evaporation. At this stage, we have constructed the edge-contact electrodes, the separations between Source (S) and drain (D) are below the resolution limit (∼20 nm-40 nm). **b)** Optical image is recorded under the dark field mode for verifying the source and drain separations which are labeled as channels. The channels down to ∼20 nm in length are fabricated by using 950K PMMA with 90 nm thickness and depositing thin contact electrodes (3/20 nm Cr/Pd). **c,d)** Overview optical images of the fabricated device. Here, we deposit 5/65 nm Cr/Au for big electrodes and contact pads. The scale bar is 20 and 500 μm, respectively.

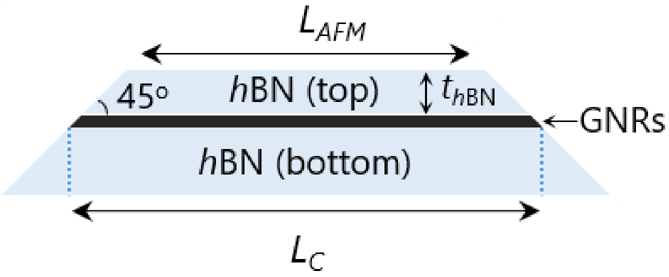

Figure S4. **Schematic cross section of the channel after the RIE process.** As shown in the schematic cross section, we estimated the actual channel length (L_C) based on the observed length (L_{AFM}) from AFM, taking into account the height of the top *h*-BN and the 45-degree angle generated by the RIE process ($L_C = L_{AFM} + 2t_{h\text{-BN}}$). The *h*-BN thickness of 7 nm and the gap from AFM of 17 nm yield a result of 31 nm for the representative device, which is close to the separation obtained from TEM.

II. ADDITIONAL MEASUREMENTS SCFETS

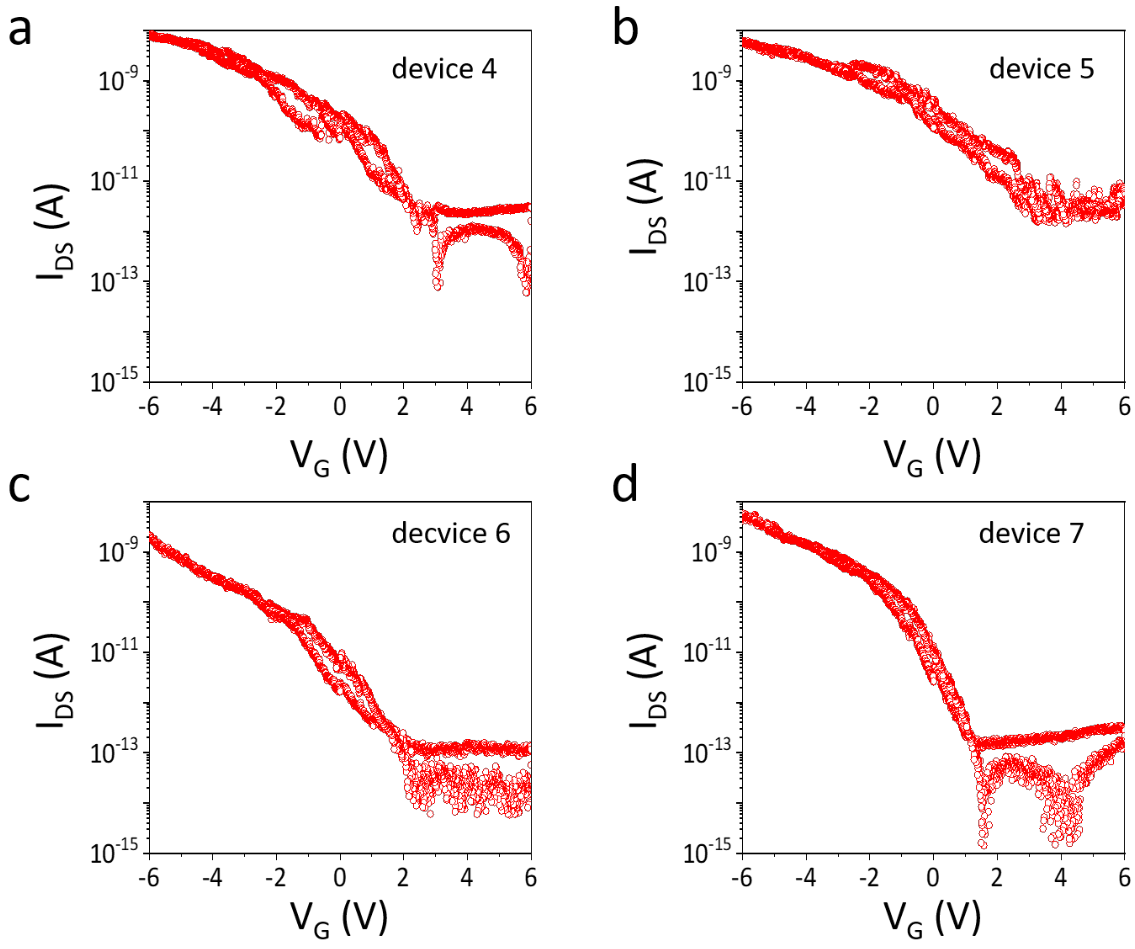

Figure S5. **Gate sweeps recorded on short channel FETs (SCFETs). a-d)** Gate sweeps (I_{DS}–V_G) obtained on four additional 9-AGNRs SCFETs recorded at 300 K.

III. SUBTHRESHOLD SWING AND FIELD-EFFECT MOBILITY EXTRACTION FOR SCFETS

To extract the Subthreshold swing, we smooth the raw data to avoid excessive noise, and then we use the SS equation, given by:[10]

$$|SS| = \frac{dV_G}{d(log_{10}|I_{(DS)}|)}, \tag{1}$$

from results, we note the effective $|SS|$ in these 3 devices low to around ∼468 mV/dec, which are comparable to the GNRs FETs with high-k dielectric gate.[11]

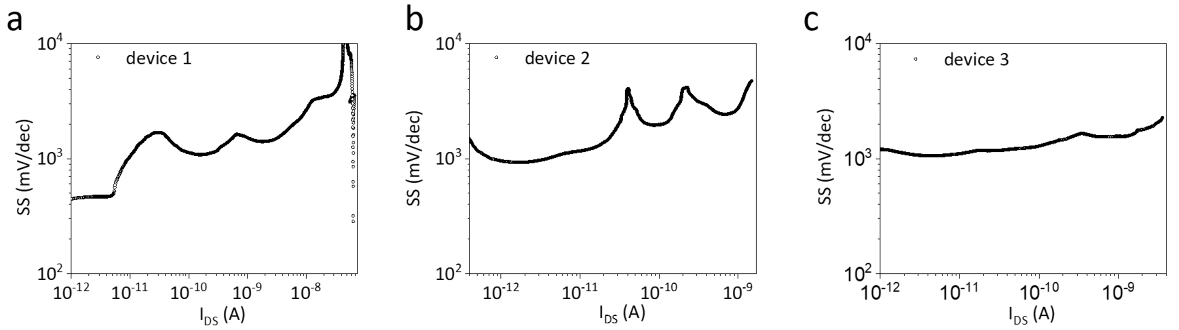

Figure S6. **Subthreshold swing extraction for SCFETs. a-c)** Subthreshold swing (SS) calculated from the I_{DS}–V_G curves for the three devices in Fig. 1f of the main text.

To extract the field-effect mobility, we use the same equation as in Ref.[10]:

$$\mu_{FE} = \frac{gL}{V_{DS}C_{OX}W}, \tag{2}$$

where g is the conductance, L is the channel length (average channel length is ∼30 nm), W is the channel width (∼500 nm) and $C_{OX} = \varepsilon\varepsilon_0/t_{ox}$. Here, the t_{ox} is the thickness of the bottom *h*-BN (∼22 nm), the relative permittivity of *h*-BN $\varepsilon = 3.8$,[12] and the vacuum permittivity $\varepsilon_0 = 8.854\times10^{-12}$ F/m. We estimate the highest μ_{FE} in our 9-AGNRs SCFETs is around ∼0.08 $cm^2v^{-1}s^{-1}$, which is more than one and three orders of magnitude higher than reported in Ref.[10] (∼10^{-3} $cm^2V^{-1}s^{-1}$) and Ref.[13] (∼10^{-5} $cm^2V^{-1}s^{-1}$), respectively. The extracted μ_{FE} is among the highest values reported in FETs where hopping as the dominant charge transport mechanism.[14] However, care should be taken as fringe currents are present in organic thin films, since the electrically contacted film (GNR film in this work) extends beyond the geometrically defined transport channel between source and drain, which may lead to the overestimation of the electron mobility.[15]

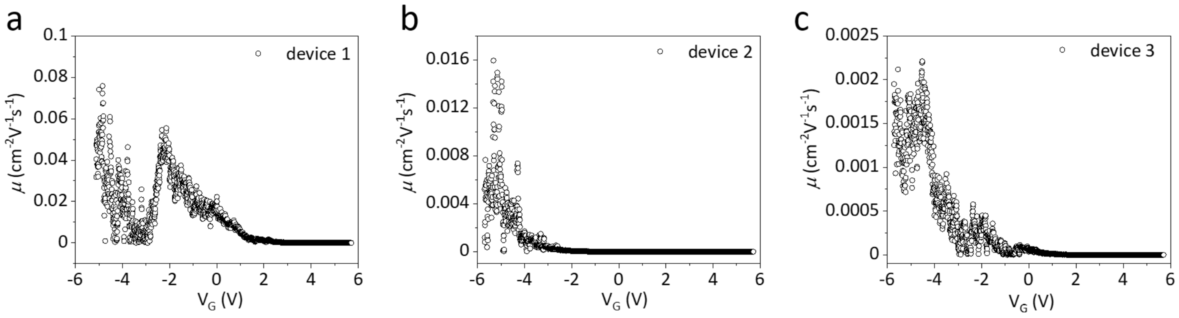

Figure S7. **Charge carrier field-effect mobility extraction for SCFETs. a-c)** Charge carrier field-effect mobility ($\mu_{\rm FE}$) extracted from $\rm I_{DS}$–$\rm V_G$ curves of three devices in Fig. 1f (main text).

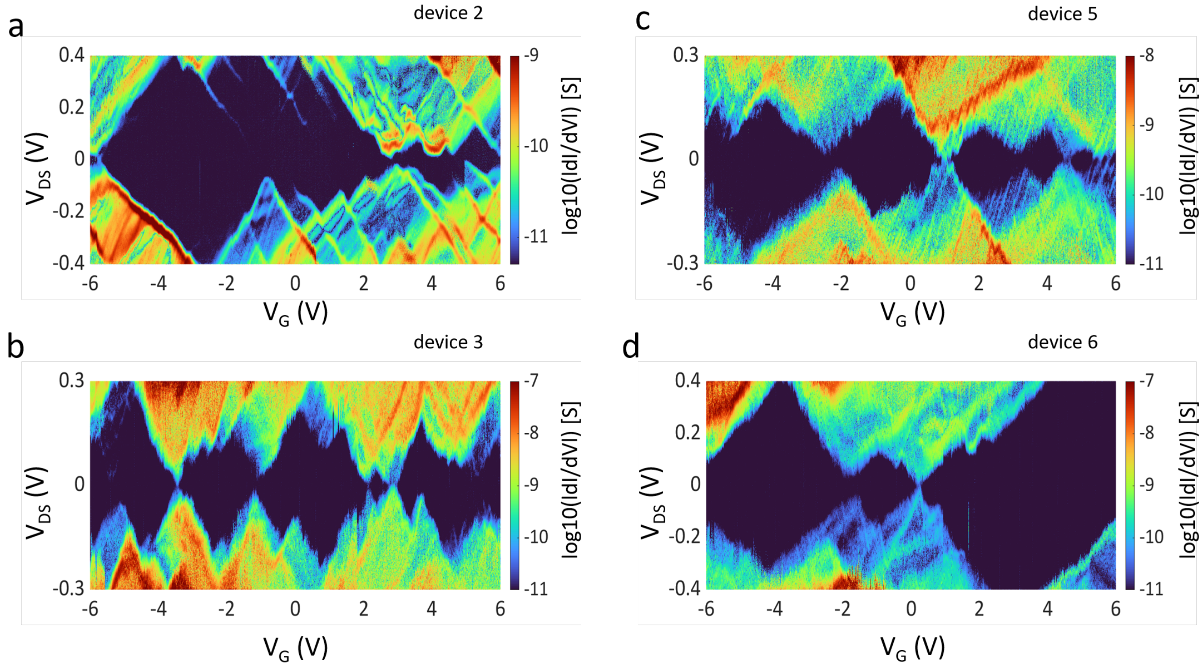

Figure S8. **Quantum dot behaviours in SCFET at 9 K. a-d)** Differential conductance ($\mathrm{d}I/\mathrm{d}V$) maps as a function of $\rm I_{DS}$ and $\rm V_G$ on logarithmic scale recorded at cryogenic temperature (9 K) on device 2, 3, 5, 6.

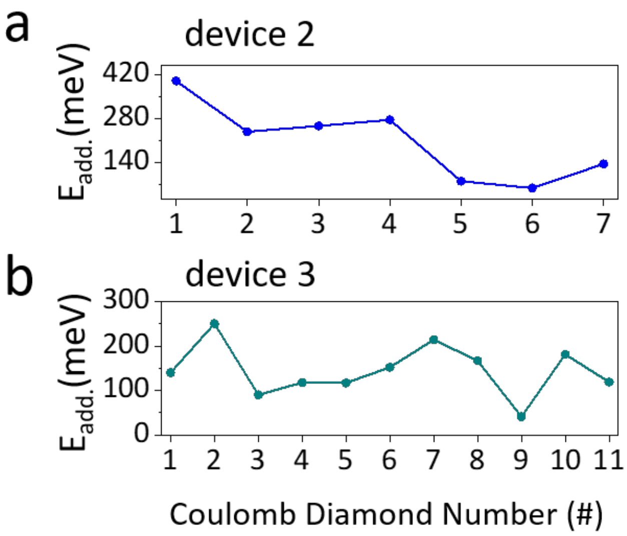

Figure S9. **Addition energy extraction. a-b)** Overview graphs of the corresponding $E_{add.}$ as a function of the Coulomb diamond number extracted from devices 2 and 3. The differential conductance ($\mathrm{d}I/\mathrm{d}V$) maps are showed in Fig. S8.

From the differential conductance maps, we extract a linecut at zero bias and fit the resonances to the Breit-Wigner (BW) model for lifetime-broadened resonant transport through a single transport level. This provides us with the tunnel coupling strength (Γ) between a single GNR and the leads. The BW model is described by:[16]

$$G(\Delta V_G) = \frac{e^2}{h}\frac{\Gamma_1\Gamma_2}{(\Delta E^2 + \frac{\Gamma^2}{4})}, \tag{3}$$

with $\Gamma = \Gamma_1 + \Gamma_2$ and the QD level detuning:

$$\Delta E = -e\alpha(\Delta V_G - V_G^{(0)}), \tag{4}$$

Here, α is the gate coupling of the gate to the QD, described using the following relation:

$$\alpha = \frac{\Delta V_{SD}}{\Delta V_G}, \tag{5}$$

where $V_G^{(0)}$ is the position of the resonance. Here, we assume that the two coupling strengths are symmetric. In total, we collect the coupling strength from 17 crossing points originating from 6 different devices (Fig. S10e). From the fit results, we find a coupling strength of 4.43 meV ± 4.22 meV, with a maximum coupling strength of about 8.65 meV. To exclude that the resonances are purely temperature broadened, we also fit our data to a thermally

broadened resonance, described as follows:[16]

$$G = \frac{e^2}{h}\frac{1}{4K_BT}\frac{\Gamma_1\Gamma_2}{\Gamma}cosh^{-2}(\frac{\Delta E}{2k_BT}), \tag{6}$$

with k_B being the Boltzmann constant and T is the bath temperature. Here, we obtain an average temperature of around ∼30 K (Fig. S10f), which is much higher than the bath temperature (9 K). This indicates that the resonance broadening observed in our measurement is the result of the combined effect of hybridization with the electrode and temperature.

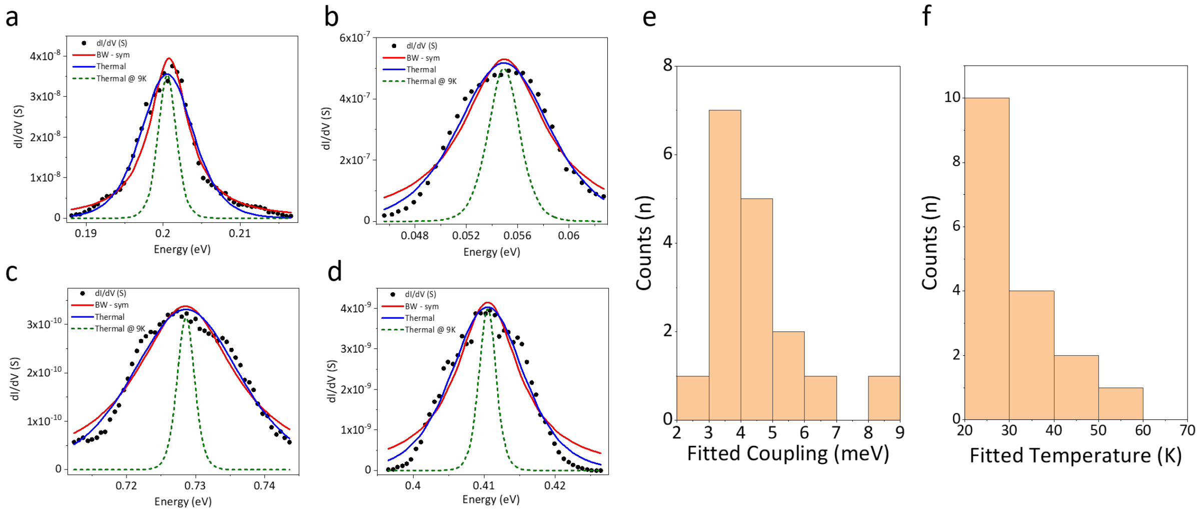

Figure S10. **Fitting of electronics coupling. a-d)** Fitting of a Coulomb peak at zero bias for four representative crossing points. The dI/dV data (black points) are extracted from the differential conductance maps. The solid red and blue lines represent a fit to the Breit-Wigner (BW) model and a thermally broadened resonance, respectively. The green line corresponds to a resonance at 9 K. e) Histogram of the coupling strengths (Γ) for all 17 crossing points obtained from the fits to the BW model. f) Histogram of the fitted temperatures for thermally broadened resonances for all 17 crossing points.

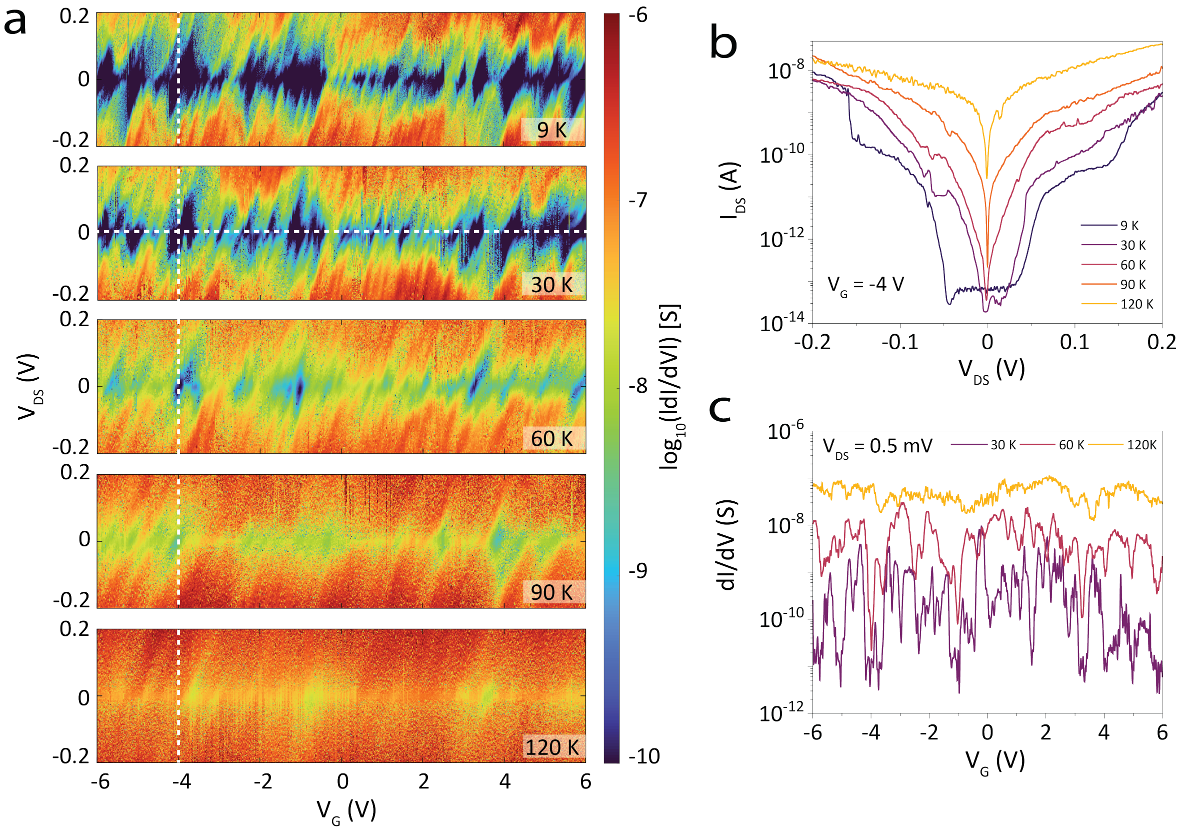

Figure S11. **Temperature-dependent transport characteristics.** **a)** Evolution of the differential conductance maps recorded on device 7 for increasing temperatures from 9 K (top) to 120 K (bottom). **b)** Bias current-voltage characteristics (I-V) along line cuts in **a)** at V_G = -4 V (Vertical dashed white line in **a)**).**c)** Differential conductance curves at three different temperatures (30 K, 60 K and 120 K) extracted from **a)** at V_{DS} = 0.5 mV (horizontal dashed white line in **a)**).

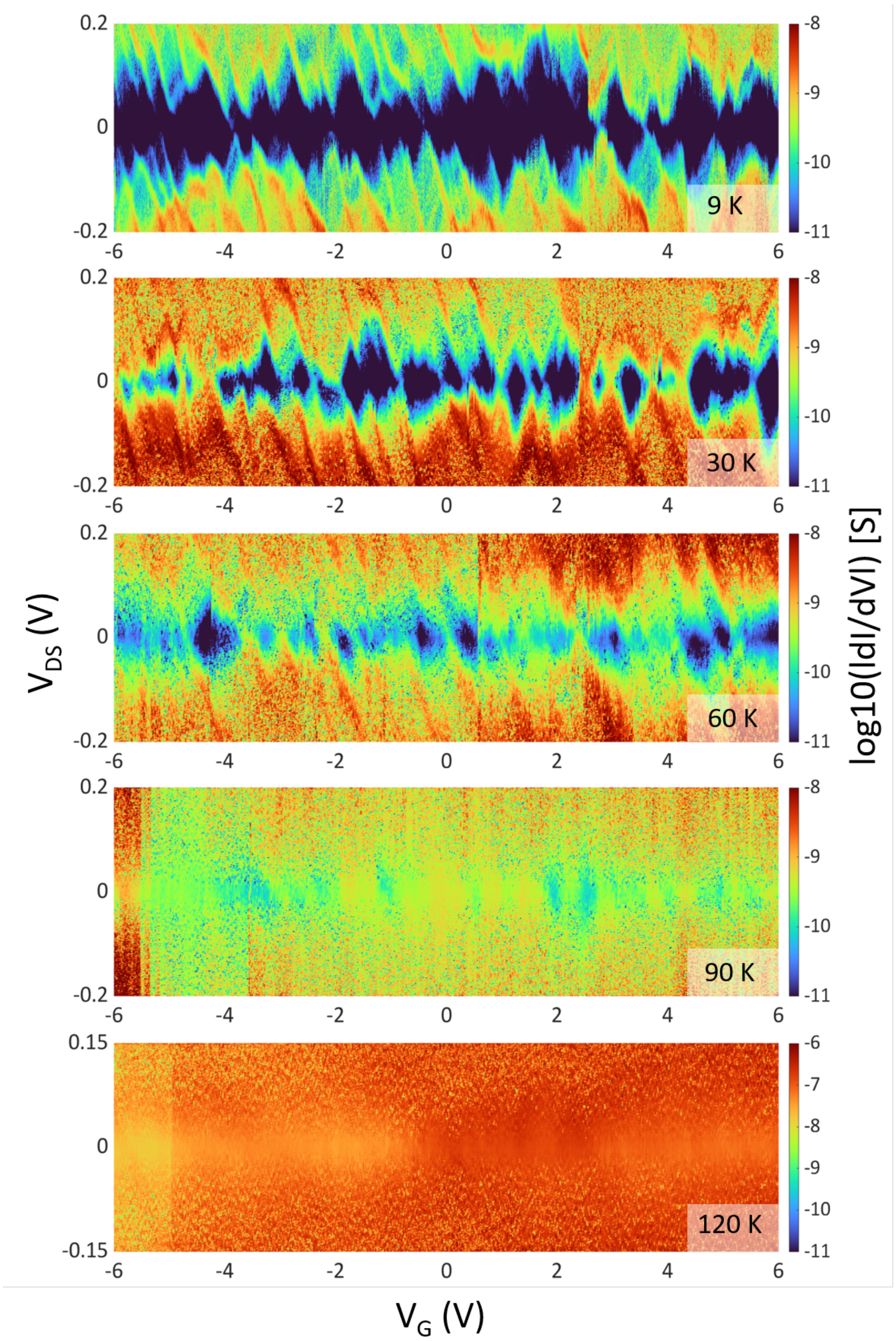

Figure S12. **Temperature-dependent differential conductance maps.** Evolution of the differential conductance maps recorded on device 8 for increasing temperatures from 9 K (top) to 120 K (bottom). The result shows a similar trend as device 7.

We perform temperature-dependent measurements on our SCFET device 7. A series of CDs is visible at a temperature of 9 K, with some CDs showing a crossing point, while others do not. And these CDs gradually smear out with increasing temperature and are

almost washed out when reaching a temperature of 120 K. The temperature-dependent I-V characteristics obtained at a fixed gate voltage of V_G = -4 V are also provided in Fig. S11. The I-V curve recorded at 9 K shows a clear gap in which transport is blocked, followed by several distinct steps. For increasing temperatures, these steps gradually become smooth and finally disappear completely when the temperature is increased up to 120 K. At the same time, we also observe that the current for all bias voltages increases as temperature goes up, predominantly in the low-bias region. A similar trend is also observed when plotting the differential conductance at low-bias versus gate voltage (numerical $\mathrm{d}I/\mathrm{d}V$ for a fixed bias voltage of V_{DS} = 0.5 mV, see Fig. S11). At temperatures below 60 K, the $\mathrm{d}I/\mathrm{d}V$ is strongly modulated by V_G and multiple Coulomb peaks are observed. However, at 120 K, the $\mathrm{d}I/\mathrm{d}V$ curve flattens out with only a small modulation being observed, while the overall conductance increases, which will be discussed in the following. We performed the same differential conductance map versus temperature measurements on device 8 and observe a similar increase in conductance around 120 K.

IV. GNR FILM GROWTH

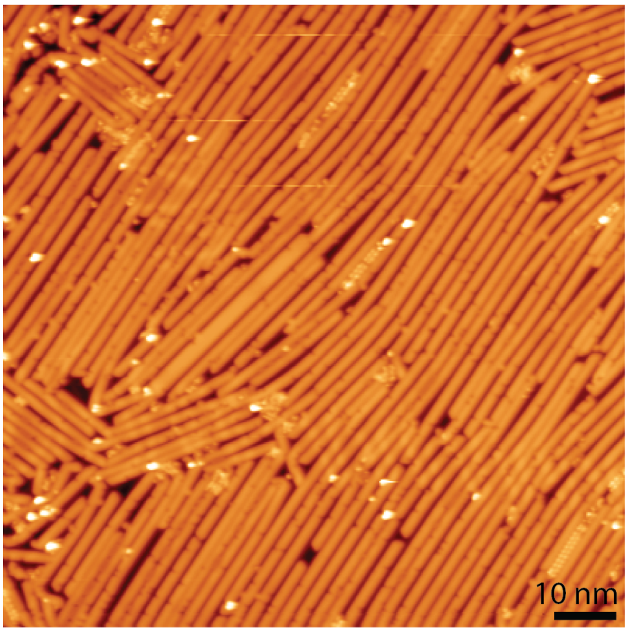

Figure S13. **Scanning tunneling microscope image of 9-AGNRs grown on Au/mica under ultra-high vacuum conditions.** The scale bar is 10 nm. The plot shows that the GNRs are grown in dense films.

V. ADDITIONAL MEASUREMENTS LCFETS

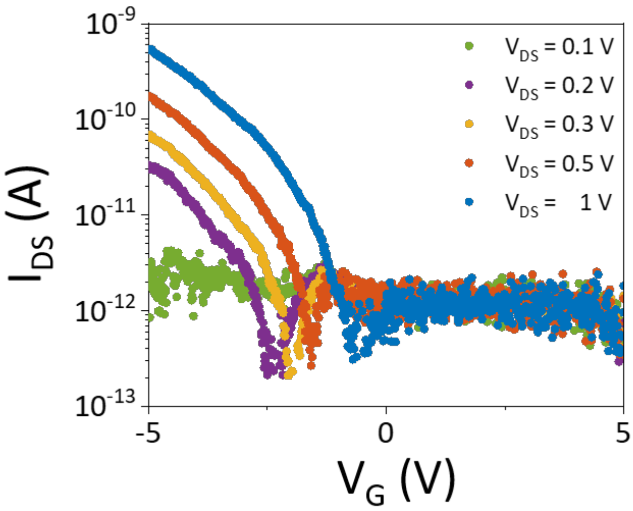

Figure S14. **Bias-dependent transport characteristics of LCFETs.** Transport characteristics of device A at various V_{DS} as a function of V_G, recorded at 300 K.

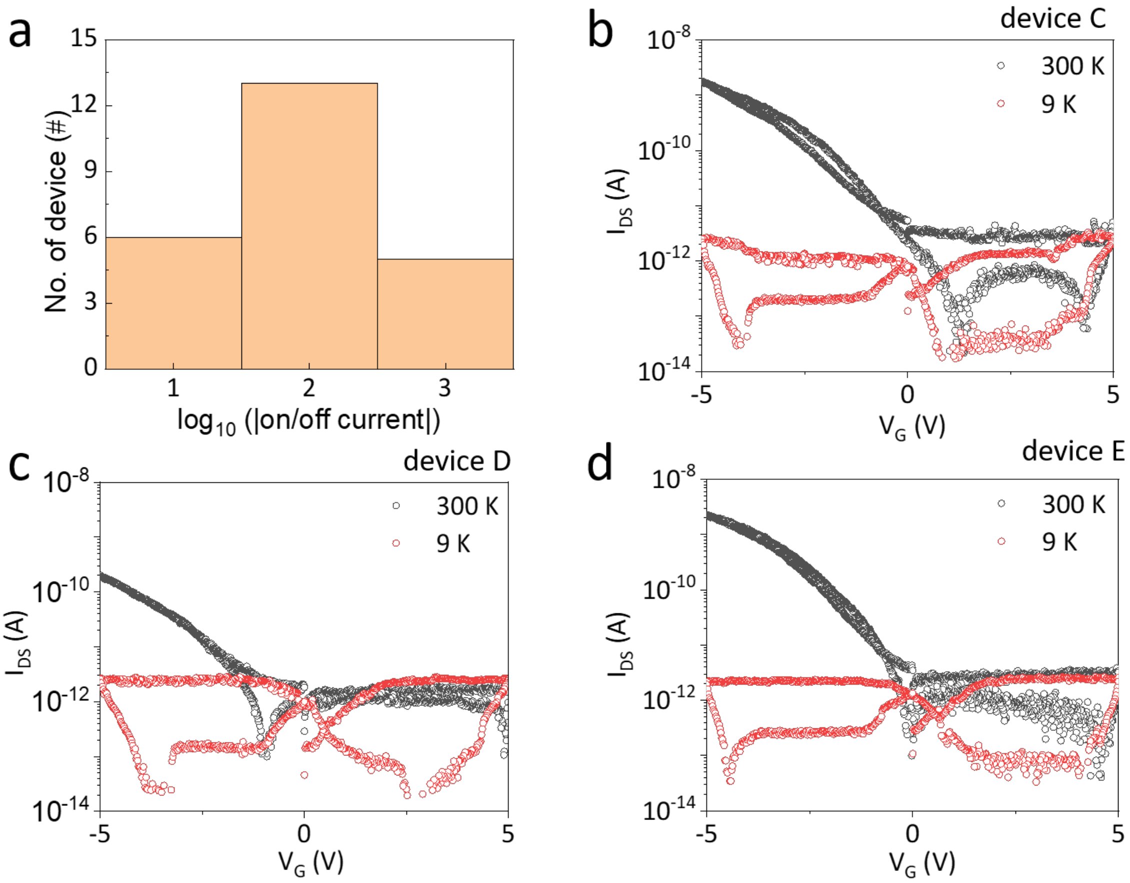

Figure S15. **Gate sweeps recorded on LCFETs. a)** Histogram of the on/off ratios of all measured 9-AGNRs film FETs (LCFETs). **b-d)** Gate sweeps (I_{DS}–V_G) recorded on three additional 9-AGNRs LCFETs T = 300 K (black) and 9 K (red). We supply V_{DS} = 1 V while sweeping V_G from 5 V to -5 V. Within the supplied gate range, the on/off ratios are in the range of 2 to 3 orders. The current drops below the measurement limit of our system at T = 9 K.

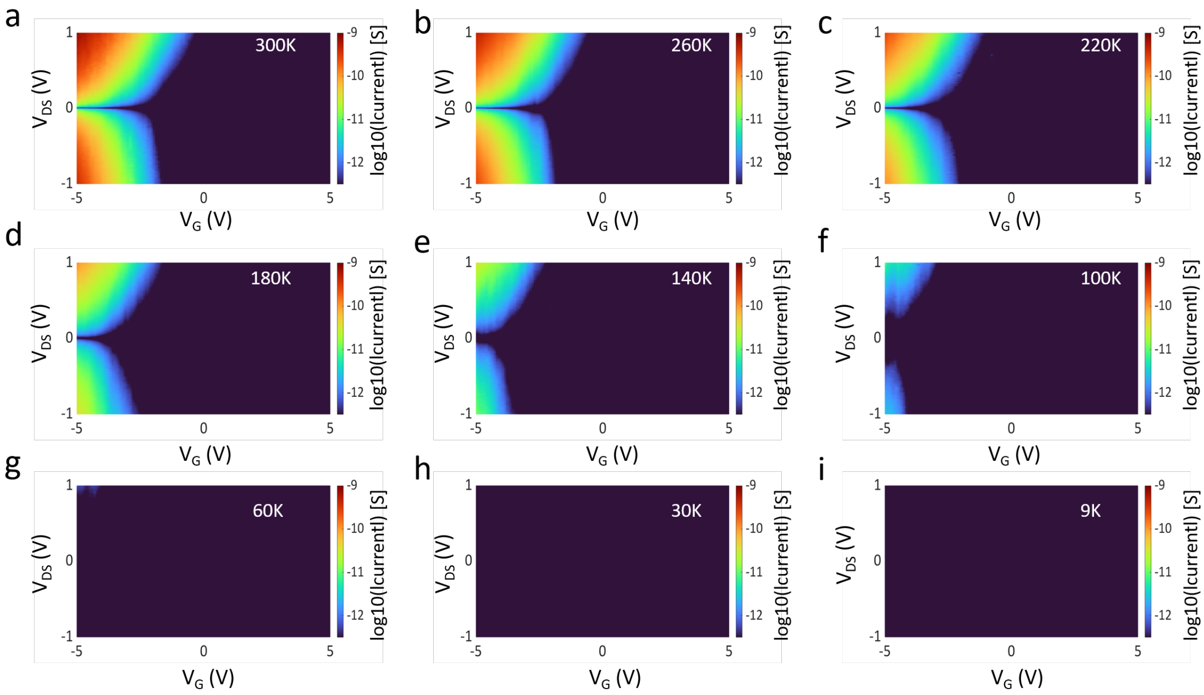

Figure S16. **Temperature-dependent current maps of device A. a-i)** Evolution of the current maps (I_{DS} versus V_{DS} and V_G) obtained for decreasing temperatures from 300 K (top left) to 9 K (bottom right) recorded on device A. With lowering the temperature, the current decreases and eventually drops below the measurement limit of our system around 60 K.

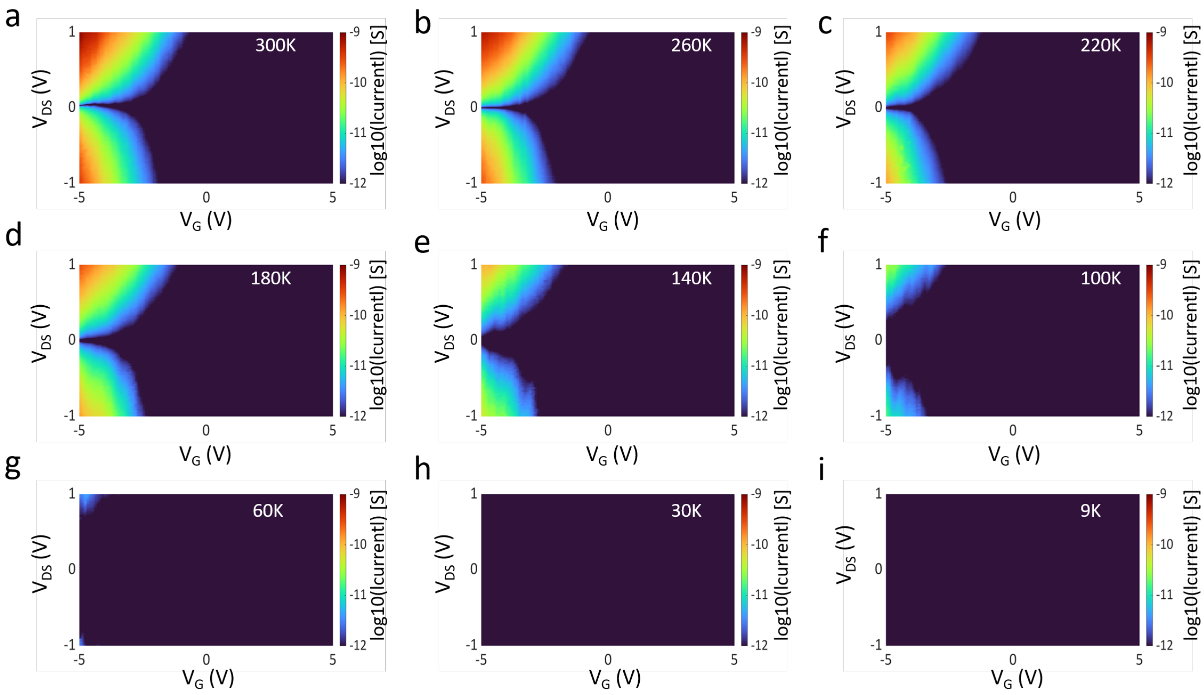

Figure S17. **Temperature-dependent current maps of device B. a-i)** Evolution of the current maps (I_{DS} versus V_{DS} and V_G) obtained for decreasing temperatures from 300 K (top left) to 9 K (bottom right) recorded on device B. With lowering the temperature, the current decreases and eventually drops below the measurement limit of our system around 60 K.

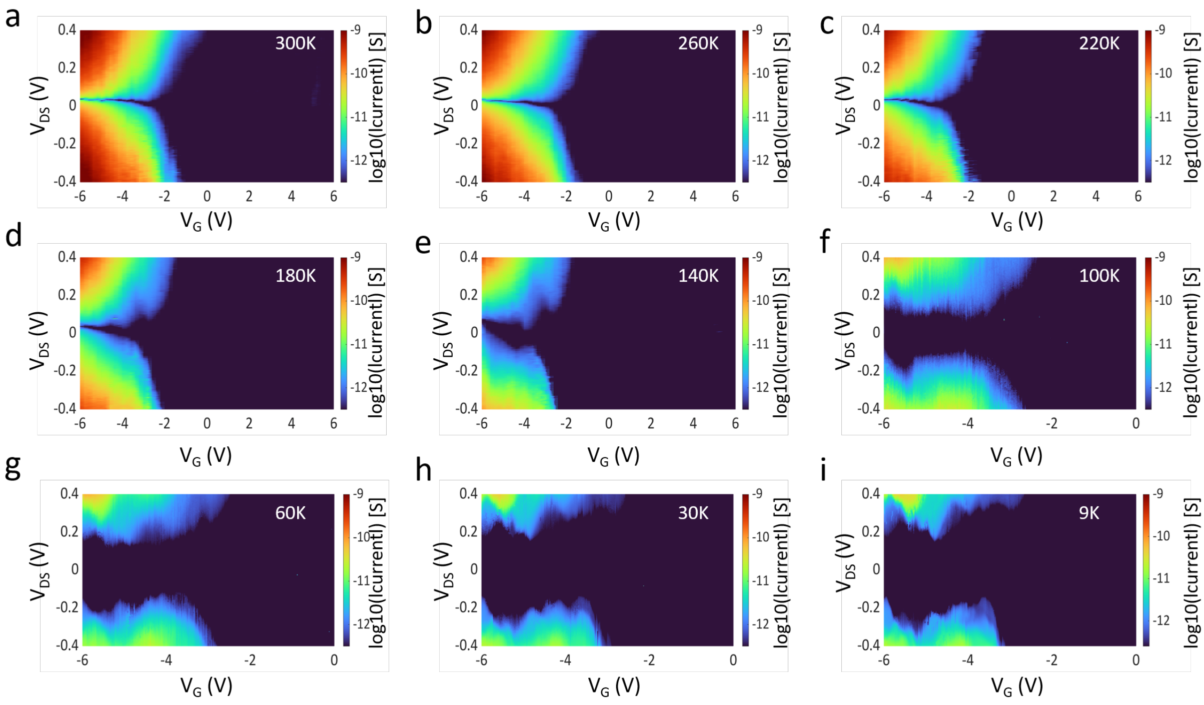

Figure S18. **Temperature-dependent current maps of device 11. a-i)** Evolution of the current maps (I_{DS} versus V_{DS} and V_G) obtained for decreasing temperatures from 300 K (top left) to 9 K (bottom right) recorded on device 9. We note that under high temperatures in the range of 300 K to 100 K, temperature-activated hopping transport is dominant, and the current decreases with decreasing temperatures. However, around T = 100 K the decay in current slows down and flattens out around T = 50 K, after which the current remains constant due to the formation of quantum dots.

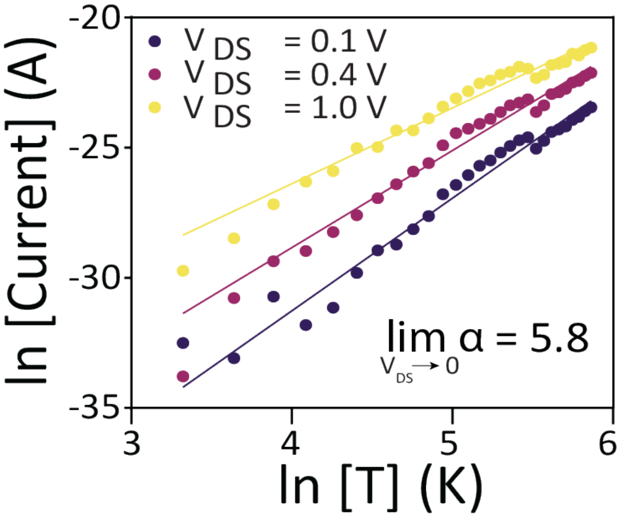

Figure S19. **Linear fit of the data to determine the scaled Kondo parameter α.** The data points represent experimental data and the solid lines are linear fits. From the slope at different bias voltages, we estimate α to be 5.8.

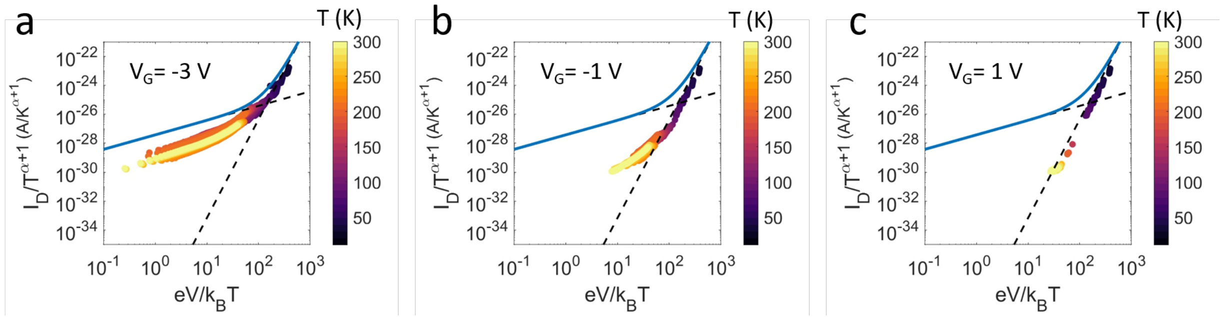

Figure S20. **Gate dependence of hopping and polaron-assisted tunneling. a-c)** For increasing gate voltages, the contribution by the temperature-activated hopping decreases until for positive gate voltages only the high-voltage regime remains. In this regime where the currents are low and therefore also the charge carrier density, polaron-assisted tunneling does not play a role anymore.

Material	Geometry type	Dielectric layer	Channel Width	On-state current	Reference
Metal	top	50 nm SiO_2	100 nm	50 nA/um @ -0.2V, -20V	Ref.[11]
Metal	top	6.5 nm HfO_2	100 nm	400 nA/um @ -0.2V, -2V	Ref.[11]
Metal	top	5 nm HfO_2	500 nm	600 nA/um @ -0.2V, -3V	Ref.[17]
Metal	top	5.5 nm HfO_2	200 nm	230 nA/um @ -0.2V, -3V	Ref.[18]
Graphene	bottom	285 nm SiO_2	400 nm	<10 nA/um @ 0.2V, -100V	Ref.[9]
Graphene	bottom	30 nm Al_2O_3	300 nm	10 nA/um @ -0.2V, 0V	Ref.[19]
Metal	edge	27 nm h-BN	500 nm	100 nA/um @ 0.2V, -6V	This work.

TABLE I. **The comparison of on-state current between this work and previous GNRs devices.**

Contact resistance is indeed very important for electronic devices, especially for GNRs devices, as it plays a critical role in their performance. However, it is a concept usually discussed in the context of 'classical' devices in which the channel material possesses a density of states that is continuous. It is therefore not straightforward to extrapolate to the 'quantum' case in which the channel possesses discrete energy levels. In the following, we will take both a 'quantum' and 'classical' view at the question.

From a quantum dot point of view, the contact resistance, and inversely the transparency of the contacts is analogous to the electronic coupling. In our devices, we find values ranging from 2.8 meV to 8.6 meV. No article published to date mentions coupling strengths for bottom-up synthesized GNR QDs. By reevaluating some of our recent results, [20] we find values in the range of 4.9 meV to 7.7 meV. Another on porphyrin GNRs states 7 meV using graphene electrodes. [21] Our values are therefore comparable with previous results using graphene and carbon nanotube electrodes.

From a 'classical' point of view, several approaches for extracting the contact resistance are reported in the literature, such as the transmission line method (TLM), four-probe measurements, and the transition voltage method. Given the extremely small size of the GNRs, the first two methods are highly challenging to perform. As our devices exhibit p-type semiconductor behavior at room temperature, the transition voltage method (TVM) should

theoretically allow us to estimate the contact resistance [22, 23] from transfer characteristics curves. However, both in our case and in reference [11] the current does not saturate at high bias and gate voltage, rendering it challenging to extract the transition voltage and saturation drain current required for extracting the contact resistance. Instead, to estimate the quality of the contact, we will compare the on-state currents at room temperature, as well as try to estimate the contact resistance at low temperatures using several assumptions.

Table 1 provides on overview of the normalized (by channel width) the on-state currents at room temperature acquired previously on GNR devices, including our work on edge-contact devices. We find that our devices, in terms of on-state currents, are comparable with some of the best top metal top-contacts devices, and better than the devices with graphene bottom-contacts. We note, however, that the number of GNRs in parallel is not known and therefore the full width of the channel is used when normalizing the current by the channel width.

We can make this estimation more accurate by analyzing the values at low temperatures, where we know that only a single GNR is contacted. This means that the effective channel width is the width of a single GNR, which is about 1nm. In addition, by making several assumptions, we can extract directly the normalized contact resistance. First, we assume that the total device resistance is given by the sum of the resistances of the connecting wires, the channel, and the two contacts: $\mathrm{R_{tot}} = \mathrm{R_{wire,left}} + \mathrm{R_{wire,right}} + \mathrm{R_{c,left}} + \mathrm{R_{c,right}} + \mathrm{R_{GNR}}$. Then, we assume that the two wire resistances ($\mathrm{R_{wire}}$) are negligible, that the contact resistances $\mathrm{R_c}$ are symmetric, and that the conductance of the channel is equal to the quantum of conductance $1\mathrm{G_0} = 1/12.9\ \mathrm{k\Omega}$. As conductance, we use the $\mathrm{d}I/\mathrm{d}V$ value along the Coulomb diamond edges. Using the above-mentioned assumptions, we find contact resistance values R_c between 0.6 MΩ and 6.2 MΩ. Given the width of the channel (GNR) of 1 nm, results in effective contact resistances between 0.6 k$\Omega\cdot\mu$m and 6.2 k$\Omega\cdot\mu$m.

[1] Zhou, J. & Dong, J. Vibrational property and raman spectrum of carbon nanoribbon. *Applied Physics Letters* **91**, 173108 (2007).

[2] Overbeck, J. *et al.* A universal length-dependent vibrational mode in graphene nanoribbons. *ACS Nano* **13**, 13083–13091 (2019).

[3] Uddin, M. A. *et al.* Improvement in electrical properties of cvd graphene on low temperature pulsed laser deposited boron nitride on sio2/si substrate. In *2015 73rd Annual Device Research Conference (DRC)*, 169–170 (IEEE, 2015).

[4] Wang, L. *et al.* One-dimensional electrical contact to a two-dimensional material. *Science* **342**, 614–617 (2013).

[5] Zomer, P., Guimarães, M., Brant, J., Tombros, N. & Van Wees, B. Fast pick up technique for high quality heterostructures of bilayer graphene and hexagonal Boron Nitride. *Applied Physics Letters* **105**, 013101 (2014).

[6] Fairbrother, A. *et al.* High vacuum synthesis and ambient stability of bottom-up graphene nanoribbons. *Nanoscale* **9**, 2785–2792 (2017).

[7] Borin Barin, G. *et al.* Surface-synthesized graphene nanoribbons for room temperature switching devices: substrate transfer and ex situ characterization. *ACS Applied Nano Materials* **2**, 2184–2192 (2019).

[8] Backes, C. *et al.* Production and processing of graphene and related materials. *2D Materials* **7**, 022001 (2020).

[9] Braun, O. *et al.* Optimized graphene electrodes for contacting graphene nanoribbons. *Carbon* **184**, 331–339 (2021).

[10] Richter, N. *et al.* Charge transport mechanism in networks of armchair graphene nanoribbons. *Scientific Reports* **10**, 1–8 (2020).

[11] Llinas, J. P. *et al.* Short-channel field-effect transistors with 9-atom and 13-atom wide graphene nanoribbons. *Nature Communications* **8**, 1–6 (2017).

[12] Laturia, A., Van de Put, M. L. & Vandenberghe, W. G. Dielectric properties of hexagonal Boron Nitride and Transition Metal Dichalcogenides: from monolayer to bulk. *npj 2D Materials and Applications* **2**, 1–7 (2018).

[13] Jeong, B. *et al.* Graphene nanoribbon field-effect transistors with top-gate polymer dielectrics.

ACS Applied Electronic Materials **4**, 2667–2671 (2022).

[14] Natali, D. & Caironi, M. Charge injection in solution-processed organic field-effect transistors: physics, models and characterization methods. *Advanced Materials* **24**, 1357–1387 (2012).

[15] Pei, K., Chen, M., Zhou, Z., Li, H. & Chan, P. K. L. Overestimation of carrier mobility in organic thin film transistors due to unaccounted fringe currents. *ACS Applied Electronic Materials* **1**, 379–388 (2019).

[16] Gramich, J., Baumgartner, A. & Schönenberger, C. Resonant and inelastic andreev tunneling observed on a carbon nanotube quantum dot. *Physical Review Letters* **115**, 216801 (2015).

[17] Lin, Y. C. *et al.* Scaling and statistics of bottom-up synthesized armchair graphene nanoribbon transistors. *Carbon* **205**, 519–526 (2023).

[18] Mutlu, Z. *et al.* Short-channel double-gate FETs with atomically precise graphene nanoribbons. In *2021 IEEE International Electron Devices Meeting (IEDM)*, 37–4 (2021).

[19] Zhang, J. *et al.* Tunable quantum dots from atomically precise graphene nanoribbons using a multi-gate architecture. *Advanced Electronic Materials* **9**, 2201204 (2023).

[20] Zhang, J. *et al.* Ultimately-scaled electrodes for contacting individual atomically-precise graphene nanoribbons (2022). 2209.04353.

[21] Chen, Z. *et al.* Phase-coherent charge transport through a porphyrin nanoribbon (2022). 2205.08499.

[22] Wang, S., Yan, Y. & Tsukagoshi, K. Transition-voltage method for estimating contact resistance in organic thin-film transistors. *IEEE Electron Device Letters* **31**, 509–511 (2010).

[23] Alghamdi, N. A. Study and analysis of simple and precise of contact resistance single-transistor extracting method for accurate analytical modeling of otfts current-voltage characteristics: Application to different organic semiconductors. *Crystals* **11**, 1448 (2021).
